# Field- and concentration-dependent relaxation of magnetic nanoparticles and optimality conditions for magnetic fluid hyperthermia

**DOI:** 10.1038/s41598-023-43140-8

**Published:** 2023-10-02

**Authors:** Patrick Ilg, Martin Kröger

**Affiliations:** 1 School of Mathematical, Physical, and Computational Sciences, University of Reading, Reading, RG6 6AX UK; 2 Magnetism and Interface Physics, Computational Polymer Physics, Department of Materials, ETH Zurich, 8093 Zurich, Switzerland

**Keywords:** Statistical physics, thermodynamics and nonlinear dynamics, Magnetic properties and materials, Nanoparticles

## Abstract

The field-dependent relaxation dynamics of suspended magnetic nanoparticles continues to present a fascinating topic of basic science that at the same time is highly relevant for several technological and biomedical applications. Renewed interest in the intriguing behavior of magnetic nanoparticles in response to external fields has at least in parts be driven by rapid advances in magnetic fluid hyperthermia research. Although a wealth of experimental, theoretical, and simulation studies have been performed in this field in recent years, several contradictory findings have so far prevented the emergence of a consistent picture. Here, we present a dynamic mean-field theory together with comprehensive computer simulations of a microscopic model system to systematically discuss the influence of several key parameters on the relaxation dynamics, such as steric and dipolar interactions, the external magnetic field strength and frequency, as well as the ratio of Brownian and Néel relaxation time. We also discuss the specific and intrinsic loss power as measures of the efficiency of magnetic fluid heating and discuss optimality conditions in terms of fluid and field parameters. Our results are helpful to reconcile contradictory findings in the literature and provide an important step towards a more consistent understanding. In addition, our findings also help to select experimental conditions that optimize magnetic fluid heating applications.

## Introduction

The relaxation dynamics of Magnetic Nanoparticles (MNPs) is of great interest in solid state and colloidal science. This field of research has received even more attention in recent years when MNPs have found numerous technological, environmental, and biomedical applications^1,2^. For example, Magnetic Fluid Hyperthermia (MFH) is currently studied intensively as a promising method for cancer treatment by field-controlled local heating of tissue with the help of MNPs^3–5^. In this method, the dissipated heat results from magnetic losses of MNP relaxation following externally applied oscillating magnetic fields^3,6,7^.

One of the main challenges today is to increase the efficiency of this method by determining optimal parameter combinations of MNPs and applied fields^3,8^. Trial and error approaches are notoriously difficult due to the large parameter space^4^. Therefore, a better understanding of the physical mechanism underlying this method is highly desirable^6,9^. While earlier studies focused on magnetic losses of individual MNPs^5,7^, many recent works consider also the effect of interactions and MNP concentration on heating efficiency^10–16^. The latter is typically measured in terms of the specific loss power (${\textrm{SLP}}$), also called specific absorption rate, which is the volumetric work done by the external field per unit cycle and magnetic mass density.

At present, there is a controversy in the literature about the concentration–dependence of the ${\textrm{SLP}}$. Several experimental studies found ${\textrm{SLP}}$ decreasing with increasing concentration of MNP (see e.g.^10–12^ and references therein). However, Martinez-Boubeta et al.^13^ instead reported non-monotonic behavior of ${\textrm{SLP}}$ with concentration, recently confirmed by Kim et al.^14^ This would suggest there might be optimal conditions for MFH in terms of MNP concentration which maximize heating efficiency^15^. Some works suggested that the different findings might be due to intrinsic properties of MNPs and cluster shapes^10,11^, the role of Brownian rotational particle motion^17,18^, or dipolar interactions^19,20^. A recent simulation study^21^ that found chain-formation to increase ${\textrm{SLP}}$ seems to support some of these suggestions, whereas a corresponding theoretical work^22^ arrives at opposite conclusions. To an extent, the confusing and sometimes contradictory findings might be related to the fact that these studies are difficult to compare, with some studies considering mobile MNPs, while other consider them immobile, and using different MNPs with different magnetic anisotropies or different coatings, adding to uncertainties in the effective dipolar interaction strengths. These differences can have pronounced effects on the heating efficiency^4,23^. Nevertheless, there seems to be agreement that MNP interactions play an important role on heating efficiency of MFH and that more research is needed to shed light on these unsolved issues^10–13,19^. It has been suggested^24,25^ that the intrinsic loss power (${\textrm{ILP}}$) is a preferable parameter compared to ${\textrm{SLP}}$ when discussing the efficiency of magnetic fluid hyperthermia from ensembles of MNPs. We will follow this suggestion here and report results for ${\textrm{ILP}}$.

**Figure 1:**
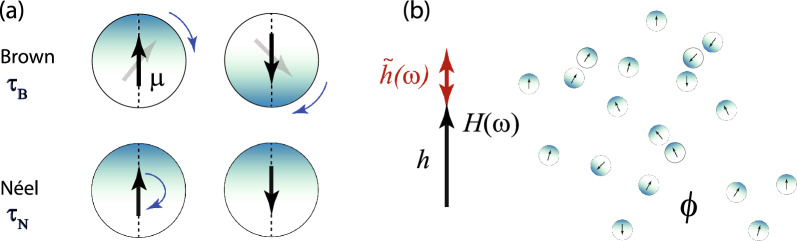
(**a**) The model system is composed of mobile and magnetically hard spherical MNPs, each characterized by diameter $\sigma$ and magnitude of the magnetic moment $\mu$, while the direction of the moment either rotates with the particle (Brownian rotational relaxation time $\tau _{\textrm{B}}$), or flips its direction inside the particle (Néel relaxation time $\tau _{\textrm{N}}$). (**b**) We study an interacting ensemble of *N* such spherical particles at volume fraction $\phi$ and temperature *T* both theoretically and numerically in the presence of an oscillating magnetic field, $\textbf{H}(\omega )$, whose strength is characterized by the time-independent Langevin parameter *h* and the magnitude ${\tilde{h}}(\omega )$ of the added small oscillating component. Our results will be discussed in terms of dimensionless parameters such as *h*, $\phi$, dipolar interaction strength $\lambda \propto \mu ^2/\sigma ^{3}k_{\textrm{B}}T$, the ratio $q=\tau _{\textrm{B}}/\tau _{\textrm{N}}$, and Langevin susceptibility $\chi _{\textrm{L}}=8\lambda \phi$.

Besides MFH, relaxation dynamics of interacting MNPs in applied fields is also an interesting topic from a theoretical point of view. Starting with the famous Landau-Lifshitz-Gilbert equation of monodomain magnetics, the field-dependent relaxation of non-interacting MNPs suspended in viscous liquids has already been studied many years ago^26–28^ and recently been revisited thanks to more efficient numerical methods^29–32^. Over the last years, considerable progress has been made to extend analytical results to moderately interacting MNPs within dynamical mean-field theory^33–37^. These theoretical results, however, rely on the rigid-dipole approximation, i.e. they neglect the internal, so-called Néel relaxation and consider Brownian particle rotation only (Fig. 1a). On the other hand, a number of simulation studies were performed for the opposite case of immobile MNPs where only Néel relaxation is present^38–40^, some studies include comparison to the corresponding experiments^41,42^. For immobile MNPs with strong magnetic anisotropy, interactions are found to lead to weaker heating efficiency, whereas the opposite is observed for MNPs with low or zero magnetic anisotropy^22,39,43^.

It is interesting to note that the case of interacting MNPs where Brownian particle rotation and internal Néel processes are both present is much less explored. It is only in the absence of external magnetic fields and particle interactions that Brownian and Néel relaxation can be considered as independent processes. In this case, the effective relaxation time $\tau _{\textrm{eff}}$ is dominated by the faster process and given by^7^

1 $$\begin{aligned} \frac{1}{\tau _{\textrm{eff}}}=\frac{1}{\tau _{\textrm{B}}} + \frac{1}{\tau _{\textrm{N}}}, \end{aligned}$$

where $\tau _{\textrm{B}}$ and $\tau _{\textrm{N}}$ denote the Brownian and Néel relaxation time, respectively. While Eq. (1) is heavily used to analyze experimental results, it should be kept in mind that applied magnetic fields or dipolar interactions lead to coupling of Brownian and Néel processes, not only invalidating Eq. (1) but also giving raise to non-exponential relaxation^44^ with corresponding non-Debye susceptibilities.^45,46^ In the presence of a static external field, it has been shown for non-interacting MNPs that the alternating current (AC) magnetic susceptibility can develop a bimodal shape where the maximum loss peak might not correspond to the longest relaxation time.^32^ Corresponding experimental studies^45^ emphasized in particular the need for improved models to better described the field-dependent Néel contribution.

Here, we investigate the field- and interaction-dependent relaxation and AC susceptibility of MNPs by comprehensive computer simulation studies and dynamic mean-field theory (Fig. 1b). Thereby, we consider the case of mobile and magnetically hard MNPs where Néel processes can be considered as rare, thermally activated events occurring alongside rotational diffusion of the nanoparticles. We have verified in our earlier work^32^ that the simplified diffusion-jump model provides a highly efficient and accurate description in this regime. From kinetic mean-field theory we obtain explicit expressions for the effective relaxation times, susceptibilities and ${\textrm{SLP}}$ as well as ${\textrm{ILP}}$. These mean-field expressions predict computer simulation results of the underlying microscopic model system rather well up to moderate dipolar interaction strengths. Our studies elucidate how dipolar interactions, the strength of a static bias field, and the ratio of Brownian and Néel relaxation time influence magnetization relaxation and the ${\textrm{SLP}}$ and ${\textrm{ILP}}$. The results obtained here also shed light on the controversy about a possible non-monotonicity of ${\textrm{SLP}}$ with concentration of MNPs and the underlying physical mechanisms. In particular, we identify different parameter regimes where ${\textrm{SLP}}$ and ${\textrm{ILP}}$ is either monotonically increasing or decreasing with concentration or attains a maximum. The latter allows us to identify optimal conditions of fluid and field parameters such that ${\textrm{ILP}}$ and ${\textrm{SLP}}$ are maximized.

## Specific and intrinsic loss power

The central quantity used to measure magnetic fluid heating efficiency is the so-called specific loss power (${\textrm{SLP}}$), also called specific absorption rate, that measures the volumetric power dissipation *P* per total mass density $\rho$ of MNPs present in the volume element in a time-varying magnetic field^5,25^,

2 $$\begin{aligned} {\textrm{SLP}}= \frac{P}{\rho }. \end{aligned}$$

In a cyclic process with angular frequency $\omega$, the volumetric power dissipation is given by $P = \frac{\omega }{2\pi }\mu _0 \oint \textbf{H}\cdot \textrm{d}\textbf{M}$ and is therefore proportional to the area enclosed by the hysteresis curve.^7^ The permeability of free space is denoted by $\mu _0$, the external magnetic field $\textbf{H}$ and the magnetization $\textbf{M}$.

We consider time-varying fields of the form $\textbf{H}(t)=\textbf{H}_0+{\tilde{\textbf{H}}}(t)$, where a harmonically oscillating field ${\tilde{\textbf{H}}}(t)={\tilde{\textbf{H}}}\cos (\omega t)$ is applied in addition to a static field $\textbf{H}_0$. If the amplitude ${\tilde{H}}=|{\tilde{\textbf{H}}}|$ is small enough to remain within the linear response regime, the resulting magnetization becomes $\textbf{M}(t)=\textbf{M}_0 + {\tilde{\textbf{M}}}(t)$, where ${\tilde{\textbf{M}}}$ is parallel to ${\tilde{\textbf{H}}}$ and can be written in terms of the in-phase ($\chi '$) and out-of-phase ($\chi ''$) component of the susceptibility as ${\tilde{\textbf{M}}}(t)={\tilde{\textbf{H}}}[\chi '\cos (\omega t) + \chi ''\sin (\omega t)]$, such that ${\textrm{SLP}}$ in Eq. (2) becomes^7^

3 $$\begin{aligned} {\textrm{SLP}}= \frac{\mu _0 \omega {\tilde{H}}^2}{2\rho } \chi ''(\omega ,h), \end{aligned}$$

where

4 $$\begin{aligned} h=\frac{\mu _0\mu |\textbf{H}_0|}{k_{\textrm{B}}T} \end{aligned}$$

denotes the dimensionless Langevin parameter associated with the static field $\textbf{H}_0$ with $\mu$ the magnitude of the magnetic moment of a single MNP and $k_{\textrm{B}}T$ the thermal energy. Setting $h=0$ in the results below corresponds to the absence of a static field, $\textbf{H}_0=0$, i.e. when only a weak oscillating field ${\tilde{\textbf{H}}}(t)$ is applied. In principle, the oscillating field ${\tilde{\textbf{H}}}$ can be applied in different directions with respect to the static field $\textbf{H}_0$. Here, we will focus on the case where these fields are applied parallel to each other.^8^ We denote the corresponding susceptibilities as $\chi _\Vert$ to distinguish them from the perpendicular case $\chi _\perp$. In the absence of a static bias field, $\textbf{H}_0=0$, the system is isotropic and $\chi _\Vert =\chi _\perp =\chi$.

Due to the appearance of the external factors $\omega$ and ${\tilde{H}}$ in Eq. (3), it has been argued that instead of ${\textrm{SLP}}$, the intrinsic loss power (${\textrm{ILP}}$) should be reported to quantify the efficiency of magnetic hyperthermia from ensembles of MNPs^25^. The ${\textrm{ILP}}$ parameter is defined as^24,25^

5 $$\begin{aligned} {\textrm{ILP}}= \frac{{\textrm{SLP}}}{\frac{\omega }{2\pi }{\tilde{H}}^2} = \frac{\pi \mu _0}{\rho }\chi _\Vert ''(\omega ,h), \end{aligned}$$

where the last equality follows from the linear response result (3). Since the dimensions of ${\textrm{ILP}}$ are $\mathrm{Hm^2/kg = m^4/(As)^2}$, we introduce the dimensionless ${\textrm{ILP}}$ by ${\textrm{ILP}}^*=\rho {\textrm{ILP}}/(\pi \mu _0\phi )$, where $\rho /\phi$ denotes the mass density of a single MNP. From the relation (5) we obtain the dimensionless

6 $$\begin{aligned} {\textrm{ILP}}^* = \frac{\chi _\Vert ''(\omega ,h)}{\phi }, \end{aligned}$$

i.e. the dimensionless ${\textrm{ILP}}^*$ is equal to the imaginary part of the susceptibility normalized with the volume fraction of magnetic material, thereby justifying the intrinsic nature of this parameter. The density of a single MNP with Fe$_2$O$_3$ core is $\rho /\phi =5175\, \mathrm{kg/m^3}$, such that we find ${\textrm{ILP}}/ {\textrm{ILP}}^* \approx 7.6\times 10^{-10}\, \mathrm{m^4/(As)^2}=0.76$ nHm$^2$/kg.

### Previous theoretical results

The central quantity governing the loss power in the linear response regime, Eqs. (3) and (5), is the out-of-phase component of the AC magnetic susceptibility. For non-interacting MNPs in the absence of externally applied fields, the complex susceptibility $\chi ^*=\chi '-i\chi ''$ is given by the Debye form

7 $$\begin{aligned} \chi _{\textrm{D}}^*(\omega ) = \frac{\chi _0}{1+i\omega \tau }, \end{aligned}$$

where $\chi _0$ denotes the zero-frequency susceptibility and $\tau$ can be identified with the effective relaxation time $\tau _{\textrm{eff}}$ defined in Eq. (1). The imaginary part $\chi _{\textrm{D}}''$ of the Debye susceptibility (7) entering ${\textrm{ILP}}$ $^*$ is given by $\chi _{\textrm{D}}''=\chi _0\omega \tau /[1+(\omega \tau )^2]$ and achieves its maximum at ${{\hat{\omega }}}=1/\tau$ where $\chi ''_{\textrm{D}}({{\hat{\omega }}})=\chi _0/2$. The corresponding result when inserting the Debye expression $\chi _{\textrm{D}}''$ into Eq. (3) or (5) is routinely used to analyze magnetic heating experiments^3,7^. For non-interacting and thermally blocked MNPs, $\chi _0$ can be identified with the Langevin susceptibility $\chi _{\textrm{L}}= n\mu _0\mu ^2/(3k_{\textrm{B}}T)$, with $n=N/V$ the number density of MNPs. Boltzmann’s constant is denoted by $k_{\textrm{B}}$. When internal Néel relaxation is included, $\chi _0$ in general depends on the magnetic anisotropy and the orientation of the easy axis of the MNPs relative to the oscillating $\textbf{H}$ field^47^.

Some authors^45^ used the empirical Havriliak-Negami model, originally proposed for dielectric spectra, to capture magnetic susceptibilities via

8 $$\begin{aligned} \chi _{\textrm{HN}}^*(\omega ) = \chi _\infty + \frac{\chi _o}{[1+(i\omega \tau )^{1-\alpha }]^\beta }, \end{aligned}$$

where $\chi _\infty$ denotes the susceptibility at infinite frequency, $\alpha , \beta$ stretching exponents and $\chi _o=\chi ^*(0)-\chi _\infty$ is related to the zero-frequency susceptibility. For the special case $\alpha =0, \beta =1$, $\chi _\infty =0$, and $\chi _o=\chi _0$, one recovers the Debye model $\chi _{\textrm{D}}^*(\omega )$. In the general case, Eq. (8) describes asymmetric and broadened spectra where the maximum of the loss peak at ${\hat{\omega }}$ is not necessarily located at $1/\tau$, i.e., ${\hat{\omega }}$ depends nonlinearly on $\tau$ and the remaining Havriliak-Negami parameters. Experimental susceptibility data could be well fitted to Eq. (8).^45^ However, due to its empirical nature, the microscopic foundation of the Havriliak-Negami model remains unclear in the present context, which makes the interpretation of the fit parameters challenging.

In the presence of a static external field, the susceptibilities and thus ${\textrm{SLP}}$ and ${\textrm{ILP}}$ depend on the orientation of the weak field oscillating relative to the static field. Within the classical model of non-interacting rigid dipoles, the AC susceptibilities for parallel and perpendicular orientations relative to the applied static field are obtained using the effective field approximation (EFA)^27^ as

9 $$\begin{aligned} \chi ^*_{\Vert }(\omega ,h)&= \chi _{\textrm{L}}\, \frac{3 L_1'(h)}{1+i\omega \tau ^{\Vert }(h)} \end{aligned}$$

10 $$\begin{aligned} \chi ^*_{\perp }(\omega ,h)&= \chi _{\textrm{L}}\, \frac{3 L_1(h)/h}{1+i\omega \tau ^{\perp }(h)} , \end{aligned}$$

with $L_1(x)=\coth (x)-1/x$ the Langevin function. The corresponding field-dependent relaxation times $\tau ^{\Vert ,\perp }(h)$ are discussed in detail later. The EFA Eqs. (9)–(10) hold only for non-interacting and thermally blocked MNPs. Recent works have shown that a modified mean-field approximation is able to extend these results to include dipolar interactions up to moderate strengths^35,48^. However, it remains unclear how Eqs. (9), (10) can be generalized beyond the rigid-dipole approximation, i.e. how Néel relaxation can be included alongside dipolar interactions. We found corrections to the Debye model due to Néel relaxation in terms of a diffusion-jump model^49^, but these results were restricted to zero external field.

In this context, it should be noted that the relaxation times $\tau$ are treated as fit parameters when Eqs. (7) and (8) are used to fit experimental data^10,45,46,50^. Below, starting from a microscopic model, we aim to derive a generalization of Eqs. (9), (10) which allows us to include the field-induced effects of Brownian as well as Néel relaxation with no adjustable parameters.

## Results of dynamic mean-field theory

In the following, we study the effects of interparticle interactions and externally applied magnetic fields on the effective relaxation behavior of a system composed of *N* spherical particles contained in a volume *V*, where each particle carries a magnetic moment $\mu \textbf{u}_i$, $i\in \{1,\ldots ,N\}$, whose orientation is encoded by the unit vector $\textbf{u}_i$. Thereby, we are especially interested in the dynamics of the magnetization $\textbf{M}$ which is defined as the total magnetic moment per unit volume, $\textbf{M}=\sum _{i=1}^N\mu \langle \textbf{u}_{i} \rangle /V=M_{\textrm{sat}}\textbf{m}$, where $M_{\textrm{sat}}=N\mu /V$ denotes the saturation magnetization and

11 $$\begin{aligned} \textbf{m}= \langle \textbf{u} \rangle = \frac{1}{N}\sum _{i=1}^{N}\langle \textbf{u}_{i} \rangle , \end{aligned}$$

a mean dimensionless magnetization per particle. Here and in the following, time-dependent configurational averages are denoted by $\langle \cdot \rangle$. Within the many-body diffusion-jump model^49^ considered here, the magnetization equation can be written as

12 $$\begin{aligned} \frac{\textrm{d}}{\textrm{d}t} \textbf{m}&= -\frac{1}{\tau _{\textrm{B}}}\textbf{m}+ \frac{1}{2\tau _{\textrm{B}}N} \sum _{i=1}^{N}\left( \langle \textbf{h}^{\textrm{loc}}_i \rangle - \langle \textbf{u}_{i}\textbf{u}_{i}\cdot \textbf{h}^{\textrm{loc}}_i \rangle \right) - \frac{1}{\tau _{\textrm{N}}N}\sum _{i,j=1}^{N}\langle \textbf{u}_{j}\, e^{-\textbf{u}_{i}\cdot \textbf{h}^{\textrm{loc}}_i} \rangle . \end{aligned}$$

Details of the model and derivation of Eq. (12) are provided in the Methods section. We note that Eq. (12) does not represent a closed-form magnetization equation as it includes higher-order correlations due to the coupling of the local field $\textbf{h}^{\textrm{loc}}_i$ to other particles, see Eq. (37). To make further analytical progress with Eq. (12), we assume the local field to be identical for all particles, $\textbf{h}^{\textrm{loc}}_i=\textbf{h}^{\textrm{loc}}$. Furthermore, we assume that cross-correlations in the Néel relaxation can be neglected. Under these assumptions, Eq. (12) simplifies to

13 $$\begin{aligned} \frac{\textrm{d}}{\textrm{d}t}\textbf{m}= - \frac{1}{\tau _{\textrm{B}}}\left[ \textbf{m}- \frac{1}{2}\left( \textbf{h}^{\textrm{loc}}- \langle \textbf{u}\textbf{u} \rangle \cdot \textbf{h}^{\textrm{loc}}\right) \right] -\frac{1}{\tau _{\textrm{N}}}\langle \textbf{u}\, e^{-\textbf{u}\cdot \textbf{h}^{\textrm{loc}}} \rangle . \end{aligned}$$

We will specify to particular mean-field approximations later. For the moment, we only assume $\textbf{h}^{\textrm{loc}}=h^{\textrm{loc}}\hat{\textbf{h}}$ with $h^{\textrm{loc}}=h^{\textrm{loc}}(h)$. Furthermore, we employ the effective field approximation (EFA) introduced by Martsenyuk *et al.*^51^ Since EFA assumes that Brownian motion randomizes dipolar orientations sufficiently, we expect this approximation to limit the results of this section to the regime $\tau _{\textrm{N}}\gg \tau _{\textrm{B}}$, and thus, $q\ll 1$, i.e. corrections to the rigid-dipole approximation. Within EFA, we find that the long-time relaxation is described by

14 $$\begin{aligned} \frac{\textrm{d}}{\textrm{d}t}\textbf{m}= - \frac{1}{\tau ^{\Vert }}(m^{\Vert }-m^{\textrm{eq}})\hat{\textbf{h}}- \frac{1}{\tau ^{\perp }} \textbf{m}^{\perp }, \end{aligned}$$

with $\hat{\textbf{h}}=\textbf{H}_0/|\textbf{H}_0|$ and the field-dependent relaxation times, $\tau ^{\Vert }$ and $\tau ^{\perp }$, parallel and perpendicular to the applied field,

15 $$\begin{aligned} \frac{1}{\tau ^{\Vert }}&= \frac{1}{\tau _{\textrm{B}}} \frac{L_{1}(h^{\textrm{loc}})}{h^{\textrm{loc}}L_{1}^{\prime }(h^{\textrm{loc}})} \frac{\textrm{d}h}{\textrm{d}h^{\textrm{loc}}} + \frac{1}{\tau _{\textrm{N}}} \frac{h^{\textrm{loc}}}{3\sinh (h^{\textrm{loc}}) L_{1}^{\prime }(h^{\textrm{loc}})} , \end{aligned}$$

16 $$\begin{aligned} \frac{1}{\tau ^{\perp }}&= \frac{1}{\tau _{\textrm{B}}} \frac{h^{\textrm{loc}}-L_{1}(h^{\textrm{loc}})}{2L_{1}(h^{\textrm{loc}})} \frac{h}{h^{\textrm{loc}}} + \frac{1}{\tau _{\textrm{N}}} \frac{(h^{\textrm{loc}})^{2}}{3\sinh (h^{\textrm{loc}})L_{1}(h^{\textrm{loc}})} . \end{aligned}$$

Details of the derivation can be found in the Methods section. Equations (15) and (16) show additivity of the field-dependent rates resulting from Brownian and Néel relaxation. These results involve the Langevin function $L_1$ defined after Eq. (4) and its derivative $L_1'(x)=x^{-2}-\sinh ^{-2}(x)$. In the absence of Néel relaxation, $\tau _{\textrm{N}}\rightarrow \infty$, these expressions for the relaxation times agree with those derived earlier^35,52^.

To arrive at more explicit expressions, we need to specify $h^{\textrm{loc}}$, i.e. choose a particular mean-field approximation. In the first order modified mean-field theory (MMF1), we have $h^{\textrm{loc}}=h + \chi _{\textrm{L}}L_{1}(h)$^53^. Within the rigid-dipole approximation, MMF1 was found to provide accurate predictions for the relaxation dynamics in case $\chi _{\textrm{L}}\lesssim 0.5$^37,54,55^. Using MMF1, explicit expressions for Eqs. (15) and (16) up to first order in $\chi _{\textrm{L}}$ are obtained, see Eqs. (49)–(54). We verified that the Brownian contributions to these expressions agree with existing results^34^. To cover also stronger dipolar interactions, a second-order modified mean-field theory (MMF2) had been proposed with^56^

17 $$\begin{aligned} h^{\textrm{loc}}= h + \chi _{\textrm{L}}L_{1}(h) + \frac{1}{16}\chi _{\textrm{L}}^{2} L_{1}(h)L_{1}'(h). \end{aligned}$$

Inserting this expression for $h^{\textrm{loc}}$ into Eqs. (15) and (16) and expanding to second order in $\chi _{\textrm{L}}$ leads to rather cumbersome expressions. For weak applied fields *h*, the relaxation times simplify to

18 $$\begin{aligned} \frac{\tau ^{\Vert ,\perp }}{\tau _{\textrm{eff}}} = 1 + \frac{\chi _{\textrm{L}}}{3(1+q)} + \frac{(1-15 q)\chi _{\textrm{L}}^2}{144(1+q)^2} + \left( c^{\Vert ,\perp }_{0} + c^{\Vert ,\perp }_{1} \chi _{\textrm{L}}+ c^{\Vert ,\perp }_{2} \chi _{\textrm{L}}^{2}\right) h^{2} + \mathcal{O}(h^{4},\chi _{\textrm{L}}^{3}), \qquad q \equiv \frac{\tau _{\textrm{B}}}{\tau _{\textrm{N}}}, \end{aligned}$$

where $\tau _{\textrm{eff}}=\tau _{\textrm{B}}/(1+q)$ defined in Eq. (1) denotes the effective single-particle relaxation time in zero field. The six coefficients $c^{\Vert ,\perp }_{k}$ appearing in Eq. (18) are given in Eqs. (55)–(57), while the *h*-independent coefficients are identical for parallel and perpendicular relaxation and reduce to $1+\chi _{\textrm{L}}/3+\mathcal{O}(\chi _{\textrm{L}}^2)$ for $q\rightarrow 0$, in agreement with existing predictions.^49^

From Eq. (18), because the $c_0$’s are negative, we see that initially $\tau ^\Vert$ decreases quadratically with increasing field strength *h*. We also note from Eq. (15) that the Néel contribution is non-monotonic in the effective field strength, i.e. the corresponding rate changes from increasing to decreasing around $h^{\textrm{loc}}\approx 2$. Consequently, for fixed *q* and sufficiently large $h^{\textrm{loc}}$, we find $\tau ^{\Vert }$ to be governed by the Brownian contribution and decrease correspondingly, while the Néel contribution freezes out.

To calculate also the dynamic magnetic susceptibility, we consider a weak, time-dependent magnetic field ${\tilde{\textbf{h}}}(t)=\mu _{0}\mu {\tilde{\textbf{H}}}(t)/k_{\textrm{B}}T$, $|{\tilde{\textbf{h}}}|\ll 1$, that is externally applied in addition to the static field $\textbf{H}_0$. Repeating the above calculations, we arrive with $\delta m^{\Vert }=m^{\Vert }-m^{\textrm{eq}}$ and ${\tilde{h}}^{\Vert }={\tilde{\textbf{h}}}\cdot \hat{\textbf{h}}$, at $(\textrm{d}/\textrm{d}t)\delta m^{\Vert }=-\delta m^{\Vert }/\tau ^{\Vert } + (\alpha _{\Vert }/3){\tilde{h}}^{\Vert }(t)$ Similarly, for the perpendicular component we find $(\textrm{d}/\textrm{d}t) \textbf{m}^{\perp }=-\textbf{m}^{\perp }/\tau ^{\perp } + (\alpha _{\perp }/3) {\tilde{\textbf{h}}}^{\perp }(t)$, from which we infer the two susceptibilities

19 $$\begin{aligned} \chi _{\Vert ,\perp } = \chi _{\textrm{L}}\frac{\tau ^{\Vert ,\perp }\alpha _{\Vert ,\perp }}{1+i\omega \tau ^{\Vert ,\perp }}, \end{aligned}$$

with amplitudes

20 $$\begin{aligned} \alpha _{\Vert }&= \frac{3L_{1}(h^{\textrm{loc}})}{\tau _{\textrm{B}}h^{\textrm{loc}}} + \frac{h^{\textrm{loc}}}{\tau _{\textrm{N}}\sinh (h^{\textrm{loc}})}, \end{aligned}$$

21 $$\begin{aligned} \alpha _{\perp }&= \frac{3(1-L_{1}(h^{\textrm{loc}})/h^{\textrm{loc}})}{2\tau _{\textrm{B}}} + \frac{h^{\textrm{loc}}}{\tau _{\textrm{N}}\sinh (h^{\textrm{loc}})}. \end{aligned}$$

In the present approximation, due to linearizing the relaxation dynamics (14) near equilibrium, the susceptibility (19) is of the Debye form and peaked at the relaxation times $\tau ^{\Vert }, \tau ^{\perp }$, respectively. Besides the location, also the height of the peak shows a dependence on the static field strength *h*. For $h\rightarrow 0$ we find isotropic behaviour with $\alpha _{\Vert }=\alpha _{\perp }=1/\tau _{\textrm{eff}}$, as expected.

The field-dependence of the Brownian contribution to the susceptibility has been calculated within EFA already some time ago^27^, at least for non-interacting MNPs. For the Néel contribution, most results in the literature are obtained for frozen particles without Brownian contribution and given (often isotropic) orientation of easy axes^27,39^. The case of mobile MNPs with Brownian and Néel contributions both present considered here has been hardly explored so far from a theoretical point of view.

With the susceptibilities at hand, we are now able to discuss the parameters ${\textrm{SLP}}$ and ${\textrm{ILP}}$. As stated above, we follow Refs^24,25^. and focus first on the parameter ${\textrm{ILP}}$. For concreteness, we specify in Eq. (6) to the case where the oscillating field is parallel to the static field. Inserting the mean-field result (19) into Eq. (6) we obtain

22 $$\begin{aligned} {\textrm{ILP}}^*= 8\lambda \tau ^\Vert \alpha _\Vert \frac{\omega \tau ^\Vert }{1+(\omega \tau ^\Vert )^2}. \end{aligned}$$

From Eq. (22), we find that the frequency dependence of ${\textrm{ILP}}^*$ follows the classical Debye law (7), i.e. first increases monotonically with frequency $\omega$ of the applied field, reaching a maximum at ${\hat{\omega }}=1/\tau ^\Vert$ before decreasing to zero for further increasing frequencies.

For weak fields and concentrations, the amplitude (20) decreases quadratically with *h* and linearly with $\chi _{\textrm{L}}$,

23 $$\begin{aligned} \tau _{\textrm{eff}}\alpha _\Vert = 1 - \frac{2+5q}{30(1+q)} \left( 1+\frac{2}{3}\chi _{\textrm{L}}+ \frac{1}{8}\chi _{\textrm{L}}^2\right) h^2 + \mathcal{O}(h^4,\chi _{\textrm{L}}^3), \end{aligned}$$

where $q\equiv \tau _{\textrm{B}}/\tau _{\textrm{N}}$, as before. In the same limit, the relaxation times are given by Eq. (18) so that we obtain from Eq. (22) for all *q* and $\omega$ and for small *h* and $\chi _{\textrm{L}}$,

24 $$\begin{aligned} {\textrm{ILP}}^*= & {} \frac{8\lambda \omega \tau _{\textrm{eff}}}{1+(\omega \tau _{\textrm{eff}})^2} \left[ 1 + \frac{2}{3(1+q)[1+(\omega \tau _{\textrm{eff}})^2]} \chi _{\textrm{L}}- \frac{q_w}{[1+(\omega \tau _{\textrm{eff}})^2]^2} \chi _{\textrm{L}}^2 \right] + \mathcal{O}(h^2,\chi _{\textrm{L}}^3) , \end{aligned}$$

25 $$\begin{aligned} q_w\equiv & {} \frac{5}{24(1+q)} [1+(\omega \tau _{\textrm{eff}})^2] - \frac{1}{9(1+q)^2}[3-(\omega \tau _{\textrm{eff}})^2]. \end{aligned}$$

where $q_w$ serves as a useful abbreviation. From Eq. (24) we find that ${\textrm{ILP}}^*$ is independent of the magnetic field strength *h* and increases initially with the dipolar interaction strength for $h\ll 1$ and $\chi _{\textrm{L}}\ll 1$. In the weak field regime where Eq. (24) holds, the dependence of ${\textrm{ILP}}^*$ on interaction strength $\chi _{\textrm{L}}$ changes, i.e. develops a maximum with respect to $\chi _{\textrm{L}}$ at

26 $$\begin{aligned} \chi _{\textrm{L}}^*= \frac{1+(\omega \tau _{\textrm{eff}})^2}{3q_w(1+q)} \end{aligned}$$

provided that $q_w>0$. This condition is satisfied either (i) for any frequency if $q>3/5$; or (ii) if $q<3/5$ (i.e. $\tau _{\textrm{eff}}/\tau _{\textrm{B}}>5/8$) and $(\omega \tau _{\textrm{eff}})^2 > (9-15q)/(23+15q)$. Thus, we expect a maximum in ${\textrm{ILP}}$ to occur as a function of dipolar interaction strength or concentration when the frequencies $\omega$ are large enough. Note, however, that ${\textrm{ILP}}^*$ decreases for frequencies larger than $\tau _{\textrm{eff}}^{-1}$, so this regime might not be very relevant for MFH applications. The other case when ${\textrm{ILP}}$ develops a maximum with concentration occurs if $\tau _{\textrm{B}}$ is as large or larger than $\tau _{\textrm{N}}$.

**Figure 2:**
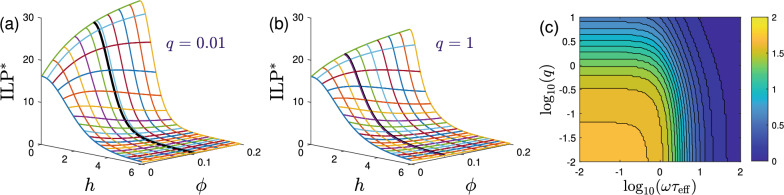
(**a**, **b**) The mean-field result (22) for the dimensionless ${\textrm{ILP}}^*$ as a function of the applied field strength *h* and volume fraction $\phi$. The remaining parameters are chosen as $\omega \tau _{\textrm{eff}}=1$, $\lambda =4$, with $q=0.01$ in panel (**a**) and $q=1$ in panel (**b**). The solid black line indicates $\phi ^*\equiv \chi _{\textrm{L}}^*/8\lambda$, where $\chi _{\textrm{L}}^*$ is given in Eq. (26). (**c**) The mean-field prediction for the critical field strength $h_c$ versus $\omega \tau _{\textrm{eff}}$ and *q*. For $h<h_c$, the ${\textrm{ILP}}^*$ initially increases with MNP concentration, whereas the opposite is the case for $h>h_c$. The analytic approximation (59) reproduces the exact mean-field result for $h_c$ qualitatively, and quantitatively for $\omega \tau _{\textrm{eff}}>1$. Contour lines equidistantly spaced at $\Delta h_c=0.1$ intervals up to $h_c=1.7$.

Figure 2a,b illustrates the mean-field result (22) for ${\textrm{ILP}}^*$ for frequency $\omega =1/\tau _{\textrm{eff}}$, dipolar interaction strength $\lambda =4$ and two values for $q=0.01$ and $q=1$. As expected, ${\textrm{ILP}}^*$ develops a maximum as a function of $\phi$ in certain cases. We note that the predicted location of the maximum, $\phi ^*=\chi _{\textrm{L}}^*/8\lambda$ with $\chi _{\textrm{L}}^*$ given by Eq. (26), is only approximate since it was derived for weak fields and small concentration. From Fig. 2a,b we furthermore find that applying a constant bias field *h* leads to a decrease of ${\textrm{ILP}}^*$. The initial quadratic decrease is worked out in the Methods section, Eq. (58). The dependence of ${\textrm{ILP}}^*$ on concentration is more involved, showing increasing as well as decreasing behavior. We define a critical field $h_c$ by $\lim _{\chi _{\textrm{L}}\rightarrow 0} d{\textrm{ILP}}^*/d\chi _{\textrm{L}}=0$, i.e. where ${\textrm{ILP}}^*$ changes from increasing to decreasing trend with interaction strength $\chi _{\textrm{L}}$ at small concentrations. Note that within mean-field theory, this critical field is independent of the dipolar interaction strength $\lambda$. Figure 2c presents a contour plot of $h_c$, showing that it tends to decrease to zero with *q* and $\omega \tau$, with its maximum $h_c\approx 1.7104$ for $q\rightarrow 0$ and $\omega \tau _{\textrm{eff}}\rightarrow 0$. Taking into account terms up to order $\mathcal{O}(h^2,\chi _{\textrm{L}})$ in ${\textrm{ILP}}^*$, we find an explicit expression for $h_c$ given by Eq. (59). For the special case $\omega \tau _{\textrm{eff}}=1$, this expression simplifies to $h_c^2 = 15/[2(5+3q)]$.

**Figure 3:**
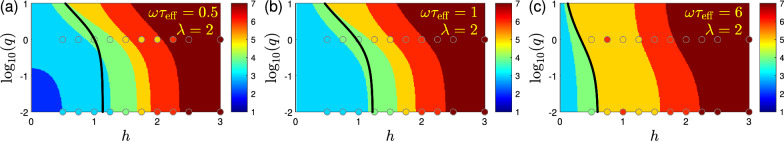
Qualitative theoretical behavior of ${\textrm{ILP}}$ $^*$ versus Langevin parameter *h* and the ratio *q*, for a particular $\lambda$ and for three different dimensionless frequencies $\omega \tau _{\textrm{eff}}$ in (**a**)–(**c**). All colors (except for circles) based on theoretical MMF2 behavior of ${\textrm{ILP}}$ $^*$ defined in Eq. (6) in $\phi \in [0,0.1]$: (1) ${\textrm{ILP}}$ $^*$ increases monotonically+convex with $\phi$, (2) increases monotonically but neither convex nor concave, (3) increases monotonically+concave, (4) goes through a maximum, (5) decreases monotonically+concave, (6) decreases monotically but neither convex nor concave, (7) decreases monotonically convex. Region (1) is absent for the present range of parameters. The green region (4) is the one which exhibits a local maximum, for all blue regions ${\textrm{ILP}}$ $^*$ increases with $\phi$. Beyond a certain critical $h=h_c$ (interfacial line between green and orange), ${\textrm{ILP}}$ $^*$ decreases with $\phi$ in any case. Black line shows our analytical approximation (59) for the critical field strength $h_c$ where the initial ${\textrm{ILP}}$ $^*$ changes from increasing to decreasing with $\phi$. The analytical expression works very well only for $h_c\ll 1$, as it was obtained via Taylor expansion about $h=0$. Colored circles mark simulation results for $q\in \{0.01,1\}$.

Figure 3 shows an overview of the different qualitative behaviors of ${\textrm{ILP}}^*(\phi )$ according to the mean-field prediction (22). For different values of the dipolar interaction strength $\lambda$ and frequencies, we show the behavior of ${\textrm{ILP}}^*$ in the plane spanned by the ratio of relaxation times *q* and the field strength *h*. We distinguish between different types of increase with $\phi$ (blue colors), existence of a maximum (green), and different forms of decreasing with $\phi$ (yellow and red colors). We observe from Fig. 3 that the ${\textrm{ILP}}^*$ behavior shows pronounced changes with *h*, but varies only weakly with *q*. We also observe that the critical field given by Eq. (59) is a good predictor for the location of a local maximum in ${\textrm{ILP}}^*$ as a function of concentration, especially at high frequencies, where $h_c$ is low enough to be captured by a fourth-order approximation. For $\omega \tau _{\textrm{eff}}=6$, for example, we find $h_c$ to be small enough such that the analytic expression (59) captures the transition from initial increase of ${\textrm{ILP}}^*$ with concentration to a decrease accurately.

We will continue the discussion of ${\textrm{ILP}}^*$ in more detail later when comparing these predictions to simulation results. But before, we first evaluate the accuracy and limits of validity of the mean-field results presented in this section via a detailed comparison to simulation results of the model system presented above.

## Simulation results and comparison to theory

Dynamic properties of the system within linear response regime are encoded in the complex magnetic susceptibility $\chi _\gamma ^*(\omega )$. Since dynamic properties are anisotropic in the presence of a static external field $\textbf{H}_0$, we distinguish parallel and perpendicular components of the susceptibility with respect to $\textbf{H}_0$. These quantities can be computed by the formula

27 $$\begin{aligned} \chi ^*_\gamma (\omega ) = -\chi _\gamma (0) \int _0^\infty \frac{{\dot{C}}_{\gamma }(t)}{C_{\gamma }(0)}e^{i\omega t}\textrm{d}t, \qquad \gamma \in \{\Vert ,\perp \} \end{aligned}$$

where we introduced the auto-correlation function

28 $$\begin{aligned} C_{\gamma }(t) = \langle \delta U_\gamma (t) \delta U_\gamma (0) \rangle , \end{aligned}$$

with the fluctuations $\delta U_\gamma (t)=U_\gamma (t)-\langle U_\gamma \rangle$, where $U_\gamma (t)=N^{-1}\sum _{j=1}^N u_{j,\gamma }(t)$ denotes the instantaneous $\gamma$-component of the reduced magnetization. The dot in Eq. (27) denotes the time derivative. The static (or zero-frequency) susceptibility is given by $\chi _\gamma (0)=3N\chi _{\textrm{L}}C_{\gamma }(0)$.

**Figure 4:**
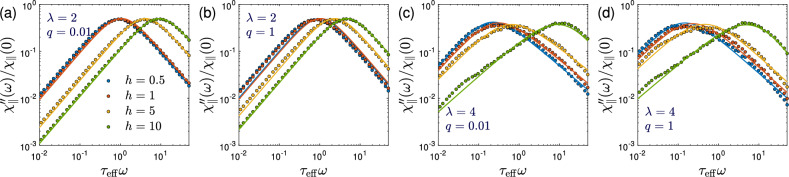
Stochastic simulations versus theory at volume fraction $\phi =0.02$. The imaginary part of the reduced dynamic magnetic susceptibility $\chi ^*_\Vert (\omega )/\chi _\Vert (0)$ parallel to the applied field is shown as a function of reduced frequency $\omega \tau _{\textrm{eff}}$. Data for several values of the constant bias field $h\in \{0.5,1,2,10\}$ are included. Other parameters are chosen as $\lambda \in \{2,4\}$ and $q\in \{0.01,1\}$ in (**a**)–(**d**). Simulation results obtained from Eq. (27) for selected frequencies are indicated by circles. In panels (**a**) and (**b**), solid lines show the corresponding theoretical results (19) with $\tau ^\Vert$, $\alpha _\Vert$, and $h^{\textrm{loc}}$ calculated from Eqs. (15), (20), and (17). In panels (c) and (d), solid lines show fits of the simulation results to Eq. (8); fitting parameters are mentioned in the text part. Simulations were performed using $N=2000$ ($\lambda =2$) and $N=10000$ ($\lambda =4$) particles for a duration of $2\times 10^5\tau _{\textrm{B}}$.

Figure 4 shows the imaginary ($\chi _\Vert ''$) part of the complex susceptibility $\chi ^*_\Vert (\omega ) = \chi '_\Vert (\omega ) - i\chi ''_\Vert (\omega )$ parallel to the applied field. The simulation data are obtained by numerically evaluating Eq. (27) from the correlation functions $C_{\gamma }(t)$ for a range of frequencies $\omega$. The correlation functions themselves are calculated by evaluating Eq. (28) from computer simulations in the stationary state using time averages. Up to moderate dipolar interaction strengths ($\lambda \le 2$), we find that the susceptibilities are well described by a Debye form. Moreover, as seen in Fig. 4a,b, the mean-field result (19) with Eqs. (15) and (20) is found to predict the simulation data quite accurately for all field strengths and over a broad range of model parameters. We emphasize that no fitting parameter is involved in the comparison between mean-field theory and simulation results. For strong dipolar interactions, the MMF2 approximation is no longer accurate (see e.g. Fig. 9 and Refs^49,54^.) and the mean-field predictions (19) are therefore less reliable. From Fig. 4c,d we indeed find not only quantitative discrepancies, but that the dynamic magnetic susceptibility can no longer be accurately described in terms of a Debye model. Instead we find that the data can be fitted to the Havriliak-Negami model, Eq. (8). There, the stretching exponents $\alpha$ and $\beta$ describe the asymmetric and broadened shape of the susceptibility spectra. For $\lambda =4$ and $\phi =0.02$, best fits are obtained for parameter values $(\alpha ,\beta )=(0.05,0.77), (0.07,0.77), (0.13,0.78), (0.21,0.70)$ for $q=0.01$ and $h=0.5, 1, 2, 10$, respectively. And correspondingly for $q=1$ these values are $(\alpha ,\beta )= (0.09,0.83), (0.14,0.91), (0.19,0.98), (0.26,1.56)$. Thus, the values of the stretching exponent $\alpha$ are found to increase with magnetic field strength *h*, reflecting a broadening of the relaxation spectrum. Interestingly, the asymmetry parameter $\beta$ behaves differently for $q=0.01$ and $q=1$. While $\beta$ remains rather insensitive to *h* for $q=0.01$, a significant increase of $\beta$ with *h* is found for $q=1$. We also note the development of a shoulder at low frequencies for strong fields that is not captured by Eq. (8).

**Figure 5:**
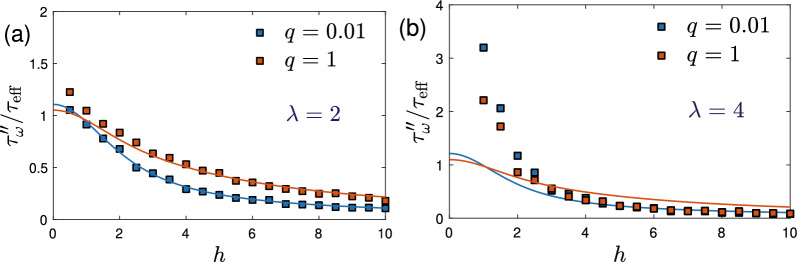
Stochastic simulations versus theory. Characteristic relaxation times $\tau _{\omega }''={\hat{\omega }}^{-1}$ obtained from the peak frequencies ${\hat{\omega }}$ of $\chi ''_\Vert$ shown in Fig. 4 normalized with the effective relaxation time $\tau _{\textrm{eff}}$ defined by Eq. (1). Data are shown as a function of applied field strength *h* for (**a**) the same parameter values as in Fig. 4, i.e., $\phi =0.02$, $\lambda =2$, and (**b**) $\phi =0.02$, $\lambda =4$. Solid lines show the corresponding mean-field predictions (15) for the long-time relaxation times. Simulations were performed using $N=2000$ ($\lambda =2$) and $N=10000$ ($\lambda =4$) particles for a duration of $2\times 10^5\tau _{\textrm{B}}$.

Next, we define characteristic relaxation times $\tau _{\omega }''=1/{\hat{\omega }}$ from the peak frequencies ${\hat{\omega }}$ of $\chi ''_\Vert$. In the parameter range investigated here, $\chi ''_\Vert$ shows a single peak so that we can obtain ${\hat{\omega }}$ unambiguously from a fit to the Debye function (7) in the vicinity of the maximum. Figure 5 shows the corresponding relaxation times $\tau _{\omega }''$ as a function of the dimensionless field strength *h*. While the field-dependent decrease of the effective relaxation times has already been studied within the rigid-dipole approximation by several authors^33–37^, our results extend these studies beyond the rigid-dipole approximation by including additional Néel relaxation. We observe from Fig. 5 that additional Néel contributions do not change qualitatively the decrease of the effective relaxation times with increasing field strength. In the absence of an external field ($h=0$), dipolar interactions increase the effective relaxation times, $\tau _{\omega }''>\tau _{\textrm{eff}}$, as already discussed by us earlier^49^ (see also Ivanov and Camp^57^ for a corresponding study in the rigid-dipole limit). It is interesting to note that the field-induced decrease of $\tau _{\omega }''/\tau _{\textrm{eff}}$ is strongest in the rigid-dipole limit $q\rightarrow 0$ and that larger values of the ratio $q=\tau _{\textrm{B}}/\tau _{\textrm{N}}$ lead to a more gradual decrease of the relaxation times with *h*. We also point out that for $\lambda =2$, the mean-field approximation (15) provides accurate predictions for $\tau _{\omega }''$ except for weak fields and $q=1$ where Eq. (15) underestimates the relaxation times. In the rigid-dipole limit, a modified Weiss mean-field approach was found to be more accurate than modified mean-field approximation for the relaxation dynamics^57^. It will be interesting for future work to explore the accuracy of such an approach for field-dependent relaxation times beyond the rigid-dipole approximation. For $\lambda =4$, the MMF2 approximation is no longer applicable and the corresponding mean-field result (15) seriously underestimates $\tau _{\omega }''$ for weak fields ($h\lesssim 2$).

**Figure 6:**
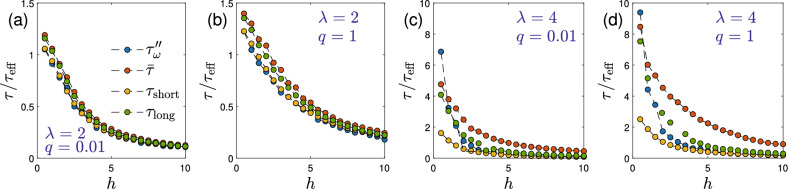
Stochastic simulation results at volume fraction $\phi =0.02$. Comparison of different relaxation times, $\tau _{\omega }''$, ${\bar{\tau }}$, $\tau _{\textrm{short}}$, $\tau _{\textrm{long}}$, defined in the text normalized with $\tau _{\textrm{eff}}$ defined by Eq. (1). Data are shown as a function of applied field strength *h* for the same parameter sets as in Fig. 4, i.e., $\lambda \in \{2,4\}$ and $q\in \{0.01,1\}$ (parameters mentioned in panels). Note the different scales on the *y*-axis. Simulations were performed using $N=2000$ ($\lambda =2$) and $N=10000$ ($\lambda =4$) particles for a duration of $2\times 10^5\tau _{\textrm{B}}$.

The deviation of the dynamic magnetic susceptibility from the Debye form reflects the non-exponential nature of the magnetization relaxation. As we have pointed out previously^32,49^, different characteristic relaxation times can be defined in this case, where the longest relaxation time does not necessarily correspond to the maximum in $\chi ''$. Therefore, Fig. 6 compares different definitions of characteristic relaxation times. In addition to $\tau _{\omega }''$ defined from the peak frequency of $\chi ''_\Vert$, we also show the integrated relaxation time, ${\bar{\tau }}=\int _0^\infty \! C_{\Vert }(t)\textrm{d}t$, a quantity that is often discussed for non-exponential relaxation^49,58^. Furthermore, we also consider the short time relaxation time $\tau _{\textrm{short}}$ obtained from the initial slope of the autocorrelation function (28), $\tau _{\textrm{short}}^{-1}=-(\textrm{d}/\textrm{d}t)\ln C_{\Vert }(t)|_{t=0}$. Finally, we also show in Fig. 6 the long-time relaxation time $\tau _{\textrm{long}}$ that governs the late stages of the relaxation. To determine $\tau _{\textrm{long}}$, we fit a double-exponential form to the autocorrelation function (28),

29 $$\begin{aligned} \frac{C_{\Vert }(t)}{C_{\Vert }(0)} = (1-c)e^{-t/\tau _{\textrm{long}}} + c\,e^{-t/\tau _1}, \end{aligned}$$

where $0\le c \le 1$ is a weight factor. Having already determined $\tau _{\textrm{short}}$, Eq. (29) contains only two fit parameters due to the relation $\tau _{\textrm{short}}^{-1}=(1-c)\tau _{\textrm{long}}^{-1}+c\tau _1^{-1}$. We use a Bayesian information criterion to decide between single– ($c=0$) and double– ($c\ne 0$) exponential fits (see Ref.^49^ for more details on this procedure). When fitting data for $\lambda =4$ to the Havriliak-Negami model, there is in principle also the fit value of the effective relaxation time $\tau$ in Eq. (8). We find this fit value to coincide with $\tau _{\omega }''$ within a factor 2–3 for these parameters. To not overburden the graph, we do not include this quantity in the comparison in Fig. 6. We observe from Fig. 6 that these different relaxation times agree with each other rather well for $\lambda =2$. Some discrepancies can be discerned for weak external fields *h*, where ${\bar{\tau }}\approx \tau _{\textrm{long}}$ are found to be somewhat larger than $\tau _{\omega }''\approx \tau _{\textrm{short}}$. For strong fields, all four definitions become indistinguishable. For strong dipolar interactions ($\lambda =4$), however, significant differences are apparent. Except for very weak fields, ${\bar{\tau }}$ is found to be the larger than the other relaxation times. In addition, for weak up to moderate field strengths, $\tau _{\textrm{long}}$ and $\tau _{\omega }''$ are found to be roughly similar to each other, while $\tau _{\textrm{short}}$ is smaller, reflecting a faster initial relaxation. For strong fields, $\tau _{\textrm{long}}$, $\tau _{\omega }''$ and $\tau _{\textrm{short}}$ become identical. Thus, besides the failure of MMF2 for strong dipolar interaction strengths, linearizing the magnetization Eq. (14) in addition misses the important distinction between $\tau _{\textrm{short}}$ and $\tau _{\textrm{long}}$.

We next turn our attention to results for the ${\textrm{ILP}}$ relevant for hyperthermia applications. Here, we limit ourselves to the linear response regime where the intrinsic loss power is given by Eq. (5). Thus, in addition to the frequency dependence shown above, we now focus on the field- and concentration dependence, as well as the influence of ratio *q* of Brownian and Néel relaxation times. To check the accuracy and limits of validity of the mean-field prediction (22), we compare this expression to our simulation results for the susceptibilities $\chi ''_\Vert$.

**Figure 7:**
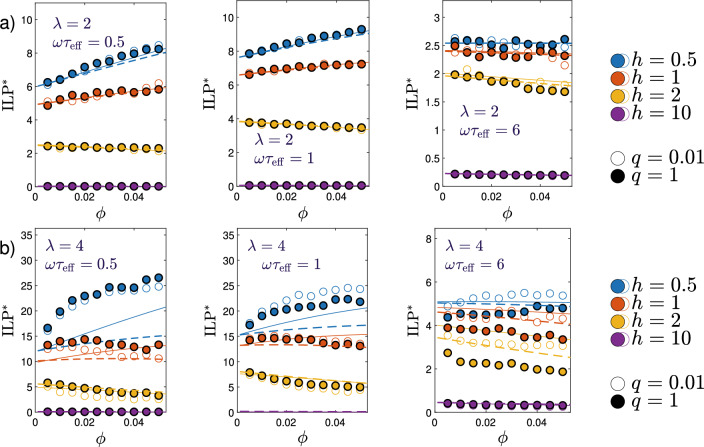
Stochastic simulations versus theory. The dimensionless intrinsic loss power ${\textrm{ILP}}$ $^*$ (6) as a function of volume fraction $\phi$, for different field strengths $h\in \{0.5,1,2,10\}$, $q=0.01$ (open symbols) and $q=1$ (filled symbols) at (**a**) $\lambda =2$ using $N=2000$ particles and (**b**) $\lambda =4$ using $N=10000$ particles (simulation: open and closed symbols, theory Eq. (22): dashed and solid lines). For both cases (**a**) and (**b**) the figure shows result at three different frequencies: $\omega \tau _{\textrm{eff}}=0.5$ (left), $\omega \tau _{\textrm{eff}}=1$ (middle), and $\omega \tau _{\textrm{eff}}=6$ (right) with $\tau _{\textrm{eff}}$ from Eq. (1).

The dimensionless ${\textrm{ILP}}^*$, Eq. (6), as a function of the volume fraction $\phi$ of MNPs is shown in Fig. 7a; the dipolar interaction strength is chosen as $\lambda =2$. Results for different field strengths *h* and different dimensionless frequencies $\omega \tau _{\textrm{eff}}$ of the external magnetic field are shown. Both of these parameters are seen to have a substantial effect on ${\textrm{SLP}}^*$. The ratio *q* of Brownian to Néel relaxation time, on the other hand, has only a minor impact on ${\textrm{ILP}}^*$, as seen by a comparison of the data for $q=0.01$ and $q=1$. Depending on the chosen parameters, we find ${\textrm{ILP}}^*$ increasing or decreasing with $\phi$, in qualitative with mean-field predictions. We also find that the mean-field prediction (22) provides a rather accurate quantitative description of the simulation data for most parameter values. In Fig. 7b, we again show ${\textrm{ILP}}^*$ as a function of $\phi$, but for stronger dipolar interactions, $\lambda =4$. Again, we compare stochastic simulation results to the mean-field prediction (22). We observe that the values of ${\textrm{ILP}}^*$ are significantly increased compared to the case $\lambda =2$ for all concentrations, field strengths and frequencies. This increase is in agreement with the mean-field prediction (22). Although strong dipolar interactions are beyond the range of validity of the MMF2 approximation, this mean-field result still describes ${\textrm{ILP}}^*$ semi-quantitatively for small concentrations or strong enough fields as long as the frequencies are not too high, $\omega \tau _{\textrm{eff}}\le 1$.

We want to emphasize that the mean-field predictions agree with the simulation results also on a more qualitative level, as summarized in Fig. 3, showing that ${\textrm{ILP}}^*$ increases with $\phi$ for small magnetic field strengths *h*, but decreases for large fields. From Fig. 7a for $\lambda =2$, $\omega \tau _{\textrm{eff}}=0.5$ and 1, we indeed find ${\textrm{ILP}}^*$ increasing with $\phi$ for $h\le 1$ and decreasing for $h\ge 2$. Similarly, from Fig. 7b for $\lambda =4$, $\omega \tau _{\textrm{eff}}=0.5$ and 1, ${\textrm{ILP}}^*$ increases with $\phi$ for $h=0.5$, goes through a maximum for $h=1$, and decreases with $\phi$ for $h\ge 2$. We note that the maxima in this regime are very shallow on the scales presented in Fig. 7.

**Figure 8:**
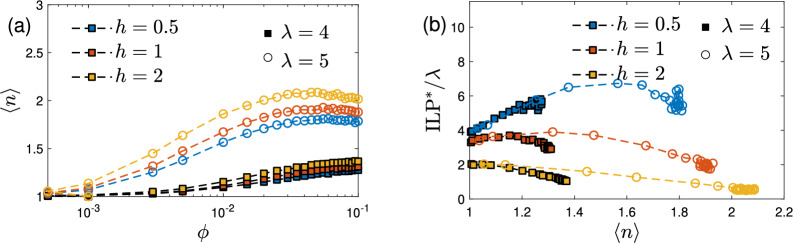
Stochastic simulation results. (**a**) Mean cluster size $\langle n\rangle$ versus volume fraction $\phi$. (**b**) Testing a proposed relation between ${\textrm{ILP}}$ $^*$ and mean cluster size^11^, shown is ${\textrm{ILP}}$ $^*/\lambda$ versus $\langle n\rangle$ as this quantity is insensitive to $\lambda$ at small $\langle n\rangle$. Parameters are chosen as $N=10000$, $q=1$, $h\in \{0.5,1,2\}$, $\omega \tau _{\textrm{eff}}=1$ and different $\phi \in [0,0.1]$ for $\lambda =4$ (filled squares) and $\lambda =5$ (open circles). The result in (**b**) is qualitatively unaffected by $\omega \tau _{\textrm{eff}}$.

Having presented our results on ${\textrm{ILP}}$, we close this section with a discussion on an alleged correlation between ${\textrm{SLP}}$ and cluster sizes^10,11^. Based on their experimental results, a phenomenological relation between ${\textrm{SLP}}$ and the mean chain size $\langle n \rangle$ of MNPs was suggested. Branquinho et al.^11^ argued that strong dipolar interactions decrease ${\textrm{SLP}}$ whereas weak interactions increase ${\textrm{SLP}}$, such that ${\textrm{SLP}}$ attains a maximum for rather short chains, $\langle n \rangle \sim 2\ldots 6$. Note that the concentration and dipolar interaction dependence of ${\textrm{SLP}}$ and ${\textrm{ILP}}$ are identical since these quantities differ only by the frequency and squared amplitude of the oscillating field, see Eq. (5). Therefore, the proposed relation ${\textrm{SLP}}(\langle n \rangle )$ should be inherited by ${\textrm{ILP}}(\langle n \rangle )$.

Here, we test this hypothesis using a cluster analysis of MNP configurations obtained from our computer simulations. We adopt a commonly employed energy criterion (see e.g.^58,59^) according to which two MNPs belong to the same cluster if their dipolar interaction energy satisfies $\Phi _{ij}^{\textrm{dd}}/k_{\textrm{B}}T\le - 1.5\,\lambda$. From analyzing many statistically independent snapshots, we calculate the average cluster size $\langle n \rangle$. In agreement with earlier results, Fig. 8a finds $\langle n \rangle$ increasing with *h*, $\phi$, and $\lambda$. For the chosen parameters, average cluster sizes are found to be rather small, $\langle n \rangle \sim 1\ldots 2$. In Fig. 8b, we plot ${\textrm{ILP}}^*/\lambda$ parametrically versus $\langle n \rangle$ for different values of the magnetic field *h* and different dipolar interaction strengths $\lambda$. While both parameters, *h* and $\lambda$, have a significant influence on ${\textrm{ILP}}$, Fig. 8b clearly shows that their combined effect is not captured by the average cluster size $\langle n \rangle$ alone, since the same value of $\langle n \rangle$ can correspond to rather different values of ${\textrm{ILP}}^*$. Note that the quantity ${\textrm{ILP}}^*/\lambda$ depends on $\langle n \rangle$ and *h*, but is approximately independent of $\lambda$ in this regime. These findings demonstrate that one has to be very careful using phenomenological arguments in terms of rigid chain-like aggregates to explain the behavior of ${\textrm{SLP}}$ or ${\textrm{ILP}}$ in this parameter regime.

## Conclusions

In this communication, we have developed a kinetic mean-field theory and performed extensive computer simulations of the dynamics of a microscopic model system that accounts for steric and dipolar interactions between MNPs, as well as their Brownian and Néel relaxation in the presence of external magnetic fields. Explicit expressions for the field-dependent relaxation times are obtained by a combination of modified mean-field theory and effective field approximation. The theoretical expressions are seen to provide a rather accurate prediction of the simulation results over a broad range of parameters, including weak up to moderate dipolar interaction strengths. Mean-field theory and simulations both show that the dynamic magnetic susceptibility and the ${\textrm{SLP}}$ and ${\textrm{ILP}}$ depend not only on the magnitude and frequency of the external magnetic field, but also on the concentration and intrinsic properties of the MNPs and their environment. Our mean-field results allow us to map out the qualitative behavior of the ${\textrm{SLP}}$ and ${\textrm{ILP}}$ over a broad range of model parameters. In particular, we explore parameter regions where the ${\textrm{ILP}}$ increases with MNP concentration, regions where ${\textrm{ILP}}$ decreases, and where a maximum in ${\textrm{ILP}}$ is expected. We have tested these theoretical predictions and overall obtained good, often quantitative, agreement with simulation results. Therefore, our findings help to maximize ${\textrm{ILP}}$ by identifying optimal conditions not only for the external magnetic field strength and frequency, but also for MNP properties, concentration and environment, via dipolar interaction strength and ratio of Brownian to Néel relaxation time. Our theoretical results become inaccurate for strong dipolar interactions where modified mean-field theory is known to break down. In this regime, we also observe deviations from Debye-like susceptibilities, pointing at the importance of non-exponential relaxation behavior. Extensions of the theory to strong dipolar interactions which are able to capture these effects are left for future work.

The presented results can be used to support several experimental findings and interpretations. In the experiments reported by Piñeiro-Redondo et al.^17^, for example, ${\textrm{SLP}}$ was reported to increase or decrease with concentration when MNPs with smaller or larger steric shells were used, respectively. Since the size of the steric shell affects the Brownian but not the Néel relaxation time, their observations can be rationalized with our model using different values of *q*. For the experiments reported by Kim et al.^14^, the ratio *q* is very small and frequencies higher than $1/\tau _{\textrm{eff}}$ are chosen. For these conditions, the critical field $h_c$ defined in Eq. (59) is small and since field strengths $h>h_c$ were applied by Kim et al.^14^, all their experiments in the dilute regime showed ${\textrm{SLP}}$ decreasing with increasing concentration, in qualitative agreement with our mean-field result. In the opposite limit of slow Brownian relaxation, $q\gg 1$, we find the critical field to become very low for any frequency, $h_c\sim \mathcal{O}(q^{-1/2})$. Although the approximation (EFA) we use is not quantitatively accurate in this regime, a number of experiments for quasi-immobilized MNPs indeed find ${\textrm{SLP}}$ to decrease with increasing concentration^10,60^.

Some authors have put forward a phenomenological model to explain the behavior of ${\textrm{SLP}}$ in terms chain-like structures formed by MNPs^11^. We have tested this idea using a detailed cluster analysis applied to many MNP configurations obtained in our computer simulations. In agreement with earlier results, we confirm that stronger dipolar interactions and larger field strengths lead to an increase in the effective cluster size $\langle n \rangle$ of MNPs. However, we find that the corresponding increase in ${\textrm{ILP}}$ or ${\textrm{SLP}}$ can not be explained by the change in $\langle n \rangle$ alone, strongly suggesting that the phenomenological model is too simplistic for the parameter region investigated here. A more qualitative explanation for the non-monotonic concentration-dependence of ${\textrm{SLP}}$ has been proposed^14^ based on the relative importance of various energy contributions. For large enough concentrations, these authors also assume rigid, chain-like structures to dominate the response, leading to ${\textrm{SLP}}$ decreasing with increasing concentration. For lower concentrations, incoherent modes are postulated to dominate since dipolar interactions are balanced by steric and magnetic field interactions. In this regime, it is argued that ${\textrm{SLP}}$ increases with increasing concentration. Within our study, we have not detected evidence supporting this picture. Instead, our studies reveal that ${\textrm{SLP}}$ and ${\textrm{ILP}}$ need to be considered in the wider parameter space including the external field as well as the fluid parameters.

Steric and dipolar interaction effects are of great interest in the recent literature on relaxation and power absorption of suspended MNPs in general. In view of biomedical applications such as hyperthermia, magnetic particle imaging or magnetic spectroscopy, this interest is in part driven by notorious agglomeration kinetics occurring in biological environments^61^. The vastly different time scales influencing MNP dynamics presents a major challenge for modelling and simulations. Although our model allows us to consider different degrees of MNP rotational mobility relative to internal Néel relaxation processes, further work is needed to better understand the interplay between MNP relaxation and agglomeration kinetics in biological environments.

## Methods

### Model system

We here employ the same model system that we studied earlier^49^, but now include an externally applied magnetic field. In order to make the paper self-contained, we here give a brief description of the model, referring the reader to the existing publication for more details and the original Ref^62^. for a justification of the model in the dilute, non-interacting limit.

#### Interaction potential

Our system consists of *N* interacting particles (MNPs) in a volume *V*, corresponding to a volume fraction $\phi = \pi n\sigma ^3/6$ with effective diameter $\sigma$. We denote with $\textbf{r}_{i}$ and $\varvec{\mu }_{i}=\mu \textbf{u}_{i}$ the position and magnetic moment of particle *i*, respectively, with $i=1,\ldots ,N$. The three-dimensional unit vectors $\textbf{u}_i$ denote the orientation of the magnetic moment. For simplicity, we consider monodisperse systems where the magnitude $\mu =|\varvec{\mu }_{i}|$ of the magnetic moment is identical for all particles. Generalizations to polydisperse systems are straightforward.

Interparticle interactions as well as the interaction with the external magnetic field $\textbf{H}$ are described by the potential

30 $$\begin{aligned} \Phi = - k_{\textrm{B}}T\sum _{i=1}^{N}\textbf{u}_{i}\cdot \textbf{h}+ \frac{1}{2}\sum _{i\ne j=1}^N \left( \Phi _{ij}^{\textrm{dd}} + \Phi _{ij}^{\textrm{s}}\right) , \end{aligned}$$

with $\textbf{h}=\mu _0\mu \textbf{H}/k_{\textrm{B}}T$ the dimensionless external field, and where $\Phi _{ij}^{\textrm{dd}}$ denotes the point-dipole interaction between particles *i* and *j*,

31 $$\begin{aligned} \Phi _{ij}^{\textrm{dd}} = \frac{\mu _0\mu ^2}{4\pi r_{ij}^3} \Big [\textbf{u}_i\cdot \textbf{u}_j - 3(\textbf{u}_i\cdot {\hat{\textbf{r}}}_{ij})(\textbf{u}_j\cdot {\hat{\textbf{r}}}_{ij}) \Big ]. \end{aligned}$$

The unit vector pointing from particle *j* to *i* is defined by ${\hat{\textbf{r}}}_{ij}=(\textbf{r}_{i}-\textbf{r}_{j})/r_{ij}$ with $r_{ij}=|\textbf{r}_{i}-\textbf{r}_{j}|$. The dipolar interaction parameter

32 $$\begin{aligned} \lambda =\frac{\mu _{0}\mu ^{2}}{4\pi \sigma ^{3}k_{\textrm{B}}T} \end{aligned}$$

plays an important role since it measures the strength of dipole-dipole interactions relative to thermal energy^63^. Note, that the two dimensionless parameters *h* and $\lambda$ adsorb $\mu _0$, $\mu$, $k_{\textrm{B}}T$, and *H*, while $\sigma$ sets the length unit.

The spherically symmetric contribution $\Phi _{ij}^{\textrm{s}}$ describes steric repulsion among the particles and is commonly modelled by a purely repulsive Lennard-Jones potential,

33 $$\begin{aligned} \Phi _{ij}^{\textrm{s}} = 4\varepsilon \left[ (\sigma /r_{ij})^{12}-(\sigma /r_{ij})^{6}\right] \Theta (r_{\textrm{c}}-r_{ij}), \end{aligned}$$

where $r_{\textrm{c}}=2^{1/6}\sigma$ and $\Theta (x)=1$ if $x>0$ and zero otherwise, denotes the Heaviside step function. The Lennard-Jones parameter $\varepsilon$ controls the strength of the repulsion, whereas $\sigma$ is a measure for the hydrodynamic particle diameter. In the following, we fix $\epsilon =k_{\textrm{B}}T$, such that the static properties are fully determined by the dimensionless parameters $\phi$, $\lambda$, *h*. Static properties of dipolar systems in an applied magnetic field described by Eq. (30) have been studied in the literature to great extent, as also reflected by existing reviews^64,65^.

It should be noted that the potential (30) does not include contributions from the magnetic anisotropy energy that arises when the magnetisation direction $\textbf{u}_{i}$ deviates from the particle’s easy axis^26,46^. Here, we assume that the anisotropy energy is sufficiently large so that magnetic moment and easy axis can be considered to be well-aligned. For cobalt and iron-oxide particles, for example, this condition is fulfilled for particles with magnetic core diameters larger than 5 and 12 nm, respectively.

#### Dynamics

We follow common practice in colloidal science and model the dynamics of the nanoparticles as overdamped Brownian motion in a viscous carrier with translational and rotational friction coefficients $\xi$ and $\xi _{\textrm{rot}}$, respectively^63,66^. With the *N*-particle, time-dependent probability density $F_N(\underline{\textbf{r}},\underline{\textbf{u}};t)$, where $\underline{\textbf{r}}=\{\textbf{r}_{1},\ldots ,\textbf{r}_{N}\}$, $\underline{\textbf{u}}=\{\textbf{u}_{1},\ldots ,\textbf{u}_{N}\}$, the diffusion-jump model proposed by us can be written as^49^

34 $$\begin{aligned} \frac{\partial }{\partial t}F_N = \hat{\texttt {L}}F_N + \hat{\texttt {Q}}F_N. \end{aligned}$$

The Fokker-Planck-Smoluchowski operator describes translational and rotational Brownian motion subject to the interaction potential $\Phi$ as^66^,

35 $$\begin{aligned} \hat{\texttt {L}}F_N&= \frac{1}{\xi }\sum _{i=1}^{N}\varvec{\nabla }_{i}\cdot \left[ F_N\varvec{\nabla }_{i}\Phi + k_{\textrm{B}}T\varvec{\nabla }_{i} F_N \right] + \frac{1}{\xi _{\textrm{rot}}}\sum _{i=1}^{N}\varvec{\mathscr {L}}_{i}\cdot \left[ F_N \varvec{\mathscr {L}}_{i}\Phi + k_{\textrm{B}}T\varvec{\mathscr {L}}_{i} F_N \right] . \end{aligned}$$

Here, we introduced $\varvec{\nabla }_{i}\equiv \partial /\partial \textbf{r}_{i}$ and the rotational operator $\varvec{\mathscr {L}}_{i}\equiv \textbf{u}_{i}\times \partial /\partial \textbf{u}_{i}$. The model corresponding to $\hat{\texttt {Q}}F_N =0$, known as “rigid-dipole approximation”, neglects internal Néel relaxation processes and has been studied extensively in the literature.^35,48,54,63^ We note that the model (35) corresponds to the “free-draining” approximation where hydrodynamic interactions between particles are neglected. While this approximation is routinely adopted, notable exceptions including these effects have been reported^21,67^.

As indicated above, we here go beyond the rigid-dipole approximation and include Néel relaxation for the case of large magnetic anisotropy energies, where magnetisation reversals $\textbf{u}_{i} \rightarrow -\textbf{u}_{i}$ within individual nanoparticles can be considered as thermally activated events. Assuming statistically independent reversals and employing the detailed balance condition to ensure the Boltzmann equilibrium $F_{\textrm{eq}}\sim \exp {[-\Phi /k_{\textrm{B}}T]}$ is a stationary solution to the diffusion-jump dynamics (34), we find^49^

36 $$\begin{aligned} \hat{\texttt {Q}}F_N(\underline{\textbf{r}},\underline{\textbf{u}};t) = \frac{1}{2\tau _{\textrm{N}}} \sum _{i=1}^{N} \left[ e^{\textbf{u}_{i}\cdot \textbf{h}^{\textrm{loc}}_i}F_N(\underline{\textbf{r}},\underline{\textbf{u}}^{(i)};t) - e^{-\textbf{u}_{i}\cdot \textbf{h}^{\textrm{loc}}_i}F_N(\underline{\textbf{r}},\underline{\textbf{u}};t)\right] \end{aligned}$$

with $\underline{\textbf{u}}^{(i)}=\{\textbf{u}_{1},\ldots ,\textbf{u}_{i-1},-\textbf{u}_{i},\textbf{u}_{i+1},\ldots ,\textbf{u}_{N}\}$ denoting the orientation state with the magnetic moment of particle *i* reversed. The hereby introduced dimensionless local field $\textbf{h}^{\textrm{loc}}_i$ acting on particle *i*, combining dipolar interactions as well as the Zeeman energy, is given by

37 $$\begin{aligned} \textbf{h}^{\textrm{loc}}_i = \textbf{h}- \lambda \sum _{j (\ne i)}(\sigma /r_{ij})^{3}[\textbf{u}_{j}-3(\textbf{u}_{j}\cdot {\hat{\textbf{r}}}_{ij}){\hat{\textbf{r}}}_{ij}]. \end{aligned}$$

Note that other (e.g. Glauber-type) rates are in principle also admissible^62^ but that we found the Arrhenius rates used in Eq. (36) to be more appropriate to recover field-dependent Néel relaxation in limiting cases^32^.

We treat the bare, single-particle Néel relaxation time $\tau _{\textrm{N}}$ as input parameter of the model. Together with the single-particle Brownian rotational diffusion time, $\tau _{\textrm{B}}=\xi _{\textrm{rot}}/(2k_{\textrm{B}}T)$, they form the basic time scales of our model. The microscopic time scale $\tau _{\textrm{D}}$ related to the attempt frequency does not appear in the model since Néel processes are modelled in Eq. (36) as thermally activated events. While $\tau _{\textrm{D}}\sim 10^{-10}$s, $\tau _{\textrm{B}}$ and $\tau _{\textrm{N}}$ are typically many orders of magnitude larger. Therefore, the present scheme is much more efficient than directly coupling Brownian particle motion to the Landau-Lifshitz-Gilbert equation for modeling internal relaxation.^68^

From Eq. (34), we can write the magnetization dynamics as

38 $$\begin{aligned} \frac{\textrm{d}}{\textrm{d}t} \textbf{m}= \frac{1}{N}\sum _{j=1}^{N} \int \!\!\int \textbf{u}_{j} (\hat{\texttt {L}}+\hat{\texttt {Q}})F_N(\underline{\textbf{r}},\underline{\textbf{u}};t)\textrm{d}\underline{\textbf{r}}\,\textrm{d}\underline{\textbf{u}}. \end{aligned}$$

Inserting the explicit form of the operators $\hat{\texttt {L}}$ and $\hat{\texttt {Q}}$ from Eqs. (35) and (36) into Eq. (38) and performing partial integration, we arrive at Eq. (12).

#### Model parameters

With the thermal energy $k_{\textrm{B}}T$ setting the energy scale, the particle diameter $\sigma$ the unit length, and the Brownian rotation diffusion $\tau _{\textrm{B}}$ setting the time scale, we here summarize the dimensionless parameters that fully characterize the model under investigation.

First, we measure the dimensionless MNP concentration in terms of the volume fraction $\phi =\pi n\sigma ^3/6$, with *n* the particle number density. The dipolar interaction strength is quantified by the dimensionless parameter $\lambda$ defined by Eq. (32), while the dimensionless external magnetic field strength is given by the Langevin parameter (4). Since the model contains two basic time scales, $\tau _{\textrm{B}}$ and $\tau _{\textrm{N}}$, their dimensionless ratio,

39 $$\begin{aligned} q = \frac{\tau _{\textrm{B}}}{\tau _{\textrm{N}}}, \end{aligned}$$

is another important parameter of our model, so that $\tau _{\textrm{B}}/\tau _{\textrm{eff}}=1+q$. Below, we consider time-dependent magnetic fields oscillating with frequency $\omega$. Therefore, the final dimensionless parameter in this study is the dimensionless frequency $\omega \tau _{\textrm{eff}}$. To summarize, our model is defined by five dimensionless quantities: $\phi$, $\lambda$, *h*, *q*, and $\omega \tau _{\textrm{eff}}$.

Within mean-field theory, the parameters $\phi$ and $\lambda$ do not appear separately. Instead, the role of interactions is approximately captured by their combined effect via the Langevin susceptibility $\chi _{\textrm{L}}$ which can be written as

40 $$\begin{aligned} \chi _{\textrm{L}}=8\lambda \phi . \end{aligned}$$

### Derivation of effective relaxation times within dynamic mean-field theory

Starting point is the magnetization Eq. (13). The yet unspecified local field $\textbf{h}^{\textrm{loc}}$ is assumed to be of the form $\textbf{h}^{\textrm{loc}}=h^{\textrm{loc}}\hat{\textbf{h}}$, with $h^{\textrm{loc}}=y(h)$ and where the function *y*(*h*) depends on the particular form of the mean-field approximation and thus, in particular also on $\chi _{\textrm{L}}$.

To arrive at closed-form expressions, we use the effective field approximation (EFA) that has been found to provide quite accurate approximations for dynamic properties under various circumstances^27,52,63^. The basic assumption of EFA is that nonequilibrium moments can be calculated with the equilibrium probability density, where the external field $\textbf{h}$ is replaced by an effective field $\zeta _{\textrm{e}}\textbf{n}$,

41 $$\begin{aligned} f_{\zeta _{\textrm{e}}}(\textbf{u}) = \frac{y(\zeta _{\textrm{e}})}{4\pi \sinh (y(\zeta _{\textrm{e}}))}e^{y(\zeta _{\textrm{e}})\textbf{u}\cdot \textbf{n}}, \end{aligned}$$

where $\textbf{n}$ is a unit vector that is not necessarily parallel to $\textbf{h}$ and $\textbf{h}^{\textrm{loc}}$. However, in equilibrium, $\zeta _{\textrm{e}}=h$, $\textbf{n}=\hat{\textbf{h}}$ and Eq. (41) reduces to the mean-field equilibrium distribution with $y(h)=h^{\textrm{loc}}$ the local field.

Evaluating the right hand side of Eq. (13) with the help of Eq. (41) we find

42 $$\begin{aligned} \frac{\textrm{d}}{\textrm{d}t}\textbf{m}= & {} - \frac{1}{\tau _{\textrm{B}}}\left[ S_1\textbf{n}- \frac{2+S_2}{6}\textbf{h}^{\textrm{loc}}+ \frac{S_2}{2}(\textbf{h}^{\textrm{loc}}\cdot \textbf{n})\textbf{n}\right] - \frac{1}{\tau _{\textrm{N}}}\frac{y[\nu \cosh (\nu )-\sinh (\nu )]}{\nu ^3\sinh (y)}\varvec{\nu }, \end{aligned}$$

where $S_j=\langle P_j(\textbf{u}\cdot \textbf{n}) \rangle =L_j(y(\zeta _{\textrm{e}}))$ are the nonequilibrium orientational order parameters, $P_j(x)$ the *j*th order Legendre polynomial, and $L_1(x)=\coth (x)-1/x$ the Langevin function. In addition, we have defined $\varvec{\nu }=y(\zeta _{\textrm{e}})\textbf{n}-\textbf{h}^{\textrm{loc}}$. Equation (42) provides a closed but nonlinear magnetization equation that can be rewritten as a time evolution equation for the effective field $\zeta _{\textrm{e}}\textbf{n}$.

Here, instead, we are interested in the long-time relaxation and therefore linearize Eq. (42) around the stationary state for constant *h*. To do this, we introduce the dimensionless deviation of the effective field from the applied field, $\varvec{\Lambda }=\zeta _{\textrm{e}}\textbf{n}-\textbf{h}$, as well as its parallel, $\Lambda ^\Vert =\varvec{\Lambda }\cdot \hat{\textbf{h}}$, and perpendicular, $\varvec{\Lambda }^\perp =\varvec{\Lambda }-\Lambda ^\Vert \hat{\textbf{h}}$, component. To first order in $\varvec{\Lambda }$ we find $\zeta _{\textrm{e}}=h+\Lambda ^\Vert$ and $\textbf{n}=\hat{\textbf{h}}+ h^{-1}\varvec{\Lambda }^\perp$. Thus, $S_j=L_j(y(h+\Lambda ^\Vert ))=S_j^{\textrm{eq}} + L_j'(h^{\textrm{loc}})(\textrm{d}h^{\textrm{loc}}/\textrm{d}h)\Lambda ^\Vert + \mathcal{O}(\Lambda ^{\Vert 2})$, where $S_j^{\textrm{eq}}=L_j(h^{\textrm{loc}})$. Furthermore, $\varvec{\nu }=({\textrm{d}}h^{\textrm{loc}}/{\textrm{d}}h)\Lambda ^\Vert {\hat{\textbf{h}}} + (h^{\textrm{loc}}/h)\varvec{\Lambda }^\perp + \mathcal{O}(\varvec{\Lambda }^2)$.

Inserting these expressions into Eq. (42) and linearizing in $\varvec{\Lambda }$, we find that the relaxation equations for the magnetization component parallel ($m^\Vert =\textbf{m}\cdot \hat{\textbf{h}}$) and perpendicular ($\textbf{m}^\perp =\textbf{m}-m^\Vert \hat{\textbf{h}}$) to the applied field separate,

43 $$\begin{aligned} \frac{\textrm{d}}{\textrm{d}t}m^\Vert&= - \left[ \frac{1-S_2^{\textrm{eq}}}{3\tau _{\textrm{B}}} + \frac{h^{\textrm{loc}}(\textrm{d}h^{\textrm{loc}}/\textrm{d}h)}{3\tau _{\textrm{N}}\sinh (h^{\textrm{loc}})}\right] \Lambda ^\Vert , \end{aligned}$$

44 $$\begin{aligned} \frac{\textrm{d}}{\textrm{d}t}\textbf{m}^\perp&= - \left[ \frac{2+S_2^{\textrm{eq}}}{6\tau _{\textrm{B}}} + \frac{(h^{\textrm{loc}})^2/h}{3\tau _{\textrm{N}}\sinh (h^{\textrm{loc}})}\right] \varvec{\Lambda }^\perp . \end{aligned}$$

Finally, we express $\varvec{\Lambda }$, and thus the effective field $\zeta _{\textrm{e}}\textbf{n}=\varvec{\Lambda }+\textbf{h}$, in terms of the magnetization components, $\Lambda ^\Vert =(m^\Vert -S_1^{\textrm{eq}})/[L_1'(h^{\textrm{loc}})\textrm{d}h^{\textrm{loc}}/\textrm{d}h]$, $\varvec{\Lambda }^\perp = (h/S_1^{\textrm{eq}})\textbf{m}^\perp$, such that we arrive at the linearized magnetization relaxation Eq. (14) with $m^{\textrm{eq}}=S_1^{\textrm{eq}}=L_1(h^{\textrm{loc}})$ and effective relaxation times

45 $$\begin{aligned} \frac{1}{\tau ^\Vert }&= \frac{1-S_2^{\textrm{eq}}}{3\tau _{\textrm{B}}}\frac{\textrm{d}h/\textrm{d}h^{\textrm{loc}}}{L_1'(h^{\textrm{loc}})} + \frac{h^{\textrm{loc}}}{3\tau _{\textrm{N}}\sinh (h^{\textrm{loc}})L_1'(h^{\textrm{loc}})}, \end{aligned}$$

46 $$\begin{aligned} \frac{1}{\tau ^\perp }&= \frac{(2+S_2^{\textrm{eq}})h}{6\tau _{\textrm{B}}L_1(h^{\textrm{loc}})} + \frac{(h^{\textrm{loc}})^2}{3\tau _{\textrm{N}}\sinh (h^{\textrm{loc}})L_1(h^{\textrm{loc}})}. \end{aligned}$$

Using the relation $S_2^{\textrm{eq}}=L_2(h^{\textrm{loc}})$ with $L_2(x)=1-3L_1(x)/x$, these expressions agree with Eqs. (15) and (16) given above.

Note that the form of $h^{\textrm{loc}}$ has not been specified yet and therefore these expressions hold for arbitrary $h^{\textrm{loc}}$ in terms of *h* and $\chi _{\textrm{L}}$, in particular.

#### First-order modified mean-field approximation

Specifying to the first-order modified mean-field approximation (MMF1), $h^{\textrm{loc}}= h + \chi _{\textrm{L}}L_1(h)$, Eqs. (15) and (16) read to first order in $\chi _{\textrm{L}}$

47 $$\begin{aligned} \frac{1}{\tau ^{\Vert ,\perp }_{\textrm{MMF1}}} = \frac{1-\chi _{\textrm{L}}t_1^{\Vert ,\perp }(h)}{\tau ^{\Vert ,\perp }_{\textrm{B},0}(h)} + \frac{1-\chi _{\textrm{L}}u_1^{\Vert ,\perp }(h)}{\tau ^{\Vert ,\perp }_{\textrm{N},0}(h)}, \end{aligned}$$

where

48 $$\begin{aligned} \tau ^\Vert _{\textrm{B},0}(h)=\tau _{\textrm{B}}\frac{hL_1'(h)}{L_1(h)}, \qquad \tau ^\perp _{\textrm{B},0}(h)=\tau _{\textrm{B}}\frac{2 L_1(h)}{h-L_1(h)} \end{aligned}$$

is the classical EFA result for the parallel relaxation time in the non-interacting limit, using the rigid-dipole approximation^51^. The coefficient

49 $$\begin{aligned} t_1^\Vert (h) = \frac{L_1(h)}{h} + \frac{L_1(h)L_1''(h)}{L_1'(h)} \end{aligned}$$

is an equivalent but more compact expression of an existing result^34^, where also the expression

50 $$\begin{aligned} t_1^\perp (h) = \frac{L_1(h)[L_2(h)-L_1^2(h)]}{h-L_1(h)} + L_1'(h) \end{aligned}$$

can be found. The non-interacting limit for the Néel contribution is well known^32^,

51 $$\begin{aligned} \frac{\tau ^\Vert _{\textrm{N},0}(h)}{\tau _{\textrm{N}}} = 3\sinh (h)\frac{L_1'(h)}{h}, \qquad \frac{\tau ^\perp _{\textrm{N},0}(h)}{\tau _{\textrm{N}}} = 3\sinh (h)\frac{L_1(h)}{h^2}. \end{aligned}$$

In Eq. (47), we also introduced the coefficients

52 $$\begin{aligned} u_1^\Vert (h) = L_1^2(h) + \frac{L_1(h)L_1''(h)}{L_1'(h)}, \quad u_1^\perp (h) = L_2(h). \end{aligned}$$

Define the effective parallel and perpendicular relaxation times in the non-interacting limit by

53 $$\begin{aligned} \frac{1}{\tau _0^{\Vert ,\perp }(h)} = \frac{1}{\tau ^{\Vert ,\perp }_{\textrm{B},0}(h)} + \frac{1}{\tau ^{\Vert ,\perp }_{\textrm{N},0}(h)}. \end{aligned}$$

Within MMF1, to first order in $\chi _{\textrm{L}}$ we obtain from Eq. (47)

54 $$\begin{aligned} \tau ^{\Vert ,\perp }_{\textrm{MMF1}}(h) = \tau _0^{\Vert ,\perp }(h)\left[ 1 + \chi _{\textrm{L}}\tau _0^{\Vert ,\perp }(h)\left( \frac{t_1^{\Vert ,\perp }(h)}{\tau ^{\Vert ,\perp }_{\textrm{B},0}(h)} + \frac{u_1^{\Vert ,\perp }(h)}{\tau ^{\Vert ,\perp }_{\textrm{N},0}(h)}\right) \right] . \end{aligned}$$

#### Second-order modified mean-field approximation

For the second-order modified mean-field approximation (17), the corresponding expressions for the relaxation times up to second order in $\chi _{\textrm{L}}$ are rather cumbersome. For weak fields, the relaxation times within MMF2 up to second order in *h* and $\chi _{\textrm{L}}$ are given by Eq. (18) with coefficients

55 $$\begin{aligned} c_0^\Vert&= -\frac{4+q}{30(1+q)}, \hspace{3.3cm} c_0^\perp = -\frac{1-q}{10(1+q)} \end{aligned}$$

56 $$\begin{aligned} c_1^\Vert&= -\frac{9+(7+q)q}{45(1+q)^2}, \hspace{3cm} c_1^\perp = \frac{q(7+6q)-11}{90(1+q)^2} \end{aligned}$$

57 $$\begin{aligned} c_2^\Vert&= \frac{[14+(47-9q)q]q-144}{2160(1+q)^3}, \hspace{1.5cm} c_2^\perp = \frac{[336+(263+54q)q]q-161}{4320(1+q)^3}, \end{aligned}$$

with the ratio *q* from Eq. (39).

### Expansion of $${\textrm{ILP}}$$$$^*$$

Within EFA, the expansion of ${\textrm{ILP}}$ $^*$ given by Eq. (22) up to linear terms in $\chi _{\textrm{L}}$ and quadratic terms in *h* becomes

58 $$\begin{aligned} {\textrm{ILP}}^* = {\textrm{ILP}}^*(h=0) - \frac{8\lambda \omega \tau _{\textrm{eff}}}{30(1+q)[1+(\omega \tau _{\textrm{eff}})^2]^2}\left[ 10+7q +(2+5q)(\omega \tau _{\textrm{eff}})^2 + \frac{20}{h_c^2}\chi _{\textrm{L}}\right] h^2 + \mathcal{O}(h^4,\chi _{\textrm{L}}^2) \end{aligned}$$

where ${\textrm{ILP}}^*(h=0)$ is given by (24), and

59 $$\begin{aligned} h_c^2 = \frac{30(1+q)[1+(\omega \tau _{\textrm{eff}})^2]}{26+27q+7q^2+6(2+q)(1+2q)(\omega \tau _{\textrm{eff}})^2+(1+q)(2+5q)(\omega \tau _{\textrm{eff}})^4}. \end{aligned}$$

From this expansion, we find that applying a weak constant bias field *h* leads to a quadratic decrease of ${\textrm{ILP}}^*$. The qualitative behavior of ${\textrm{ILP}}$ $^*$ at small $\chi _{\textrm{L}}$ depends on the magnitude of *h* because $h_c$ solves $\lim _{\chi _{\textrm{L}}\rightarrow 0} d{\textrm{ILP}}^*/d\chi _{\textrm{L}}=0$. For $h>h_c$ it rises with increasing $\chi _{\textrm{L}}$, while it decreases with $\chi _{\textrm{L}}$ for $h<h_c$. The approximation (59) for $h_c$ is only useful as long as $h_c\ll 1$, which is generally the case for sufficiently large $\omega \tau _{\textrm{eff}}$. The unapproximated expression for $h_c$ is plotted in Fig. 2c.

### Simulation approach and validation

To check the quality of the mean-field approximations used in the Dynamic mean-field theory section, we perform extensive computer simulations of the model system presented in the Model system section below. The diffusion-jump model (34) can be integrated using operator-splitting techniques^49^,

60 $$\begin{aligned} F_N(t+\Delta t) = e^{\Delta t [\hat{\texttt {L}}+ \hat{\texttt {Q}}]} F_N(t) \approx e^{\Delta t \hat{\texttt {L}}}F_N + \Delta t\, \hat{\texttt {Q}}F_N, \end{aligned}$$

where we used the short-hand notation $F_N(t)=F_N(\underline{\textbf{r}},\underline{\textbf{u}};t)$. From Eq. (60) we find that the dynamics can be integrated forward over a short time step $\Delta t$ by a succession of a diffusion step $e^{\Delta t\hat{\texttt {L}}}$ and a jump process $\Delta t\, \hat{\texttt {Q}}$. Since diffusion is described by the Fokker-Planck operator (35), we use Brownian Dynamics simulations to propagate the configurations $\{\underline{\textbf{r}}(t),\underline{\textbf{u}}(t)\}$ by a time step $\Delta t$, which requires $\Delta t\ll \tau _{\textrm{B}}$.

To simulate the jump process $\Delta t\, \hat{\texttt {Q}}$ for a time interval $\Delta t$, we consider the probability $p_i$ that the moment $\textbf{u}_i$ has not reversed its orientation between $t=0$ and $t=\Delta t$. According to Eq. (36), this probability is given by $p_i=e^{-r_i \Delta t}$ with rate $r_i = e^{-\textbf{u}_i\cdot \textbf{h}^{\textrm{loc}}_i}/(2\tau _{\textrm{N}})$. Thus, the probability that the reversal of moment $\textbf{u}_i$ takes place in the time interval $\Delta t$ is $1-e^{-r_i\Delta t}$, a well-known property of Poisson processes. Therefore, calculating the probabilities $p_i(t)$ from the current configuration and resulting $\textbf{h}^{\textrm{loc}}_i(t)$, we reverse the orientations of the magnetic moments $\textbf{u}_i(t)\rightarrow -\textbf{u}_i(t)$ in the time step $\Delta t$ with probability $1-p_i(t)$.

We note that the time step $\Delta t$ should be small enough so that (i) $r_i\Delta t<1$, i.e. at most a single reversal per magnetic moment within $\Delta t$. In addition, we also require (ii) that the local field $\textbf{h}^{\textrm{loc}}_i$ can be considered constant over this time interval. This implies that the fraction $x_{\textrm{flip}}$ of magnetic moments that reverse their orientation during $\Delta t$ must be relatively small (we ensured that $x_{\textrm{flip}}$ does not exceed 0.05). For $\tau _{\textrm{N}}\gg \tau _{\textrm{B}}$ (i.e. considering small corrections to rigid-dipole approximation), the time step from stochastic simulations should in general be small enough to ensure conditions (i) and (ii) above. For $\tau _{\textrm{N}}\approx \tau _{\textrm{B}}$ or even $\tau _{\textrm{N}}<\tau _{\textrm{B}}$, condition (ii) might not be satisfied. In this case, one might re-calculate the probabilities $p_i$ whenever $x_{\textrm{flip}}$ is exceeded or perform *k* successive jump steps, each using a reduced time step $\Delta t/k$ to integrate the $\hat{\texttt {Q}}$ part of the dynamics. In the extreme case $\tau _{\textrm{N}}\ll \tau _{\textrm{B}}$ (corresponding to basically immobile particles, not considered here), such schemes become inefficient and one resorts to kinetic Monte-Carlo simulations.^39,40^

**Figure 9:**
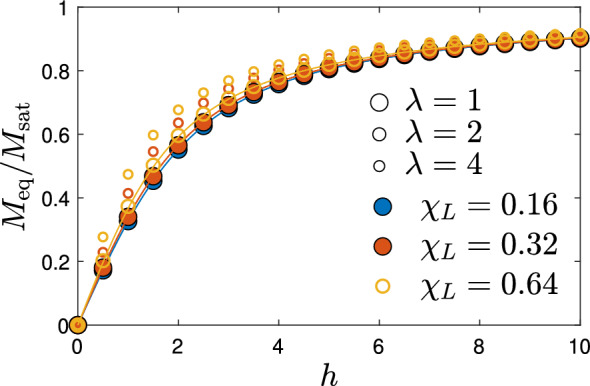
Stochastic simulation results. Normalized equilibrium magnetization $M_{\textrm{eq}}/M_{\textrm{sat}}$ as a function of applied field strength *h*. Different symbols denote different values of dipolar interaction strength $\lambda \in \{1,2,4\}$ and volume fraction $\phi \in \{0.01,0.02,0.04\}$. Solid lines correspond to the second order modified mean-field approximation, Eq. (61).

We consider systems of *N* particles in a cubic simulation box of volume *V*. The corresponding volume fraction is defined as $\phi =N\pi \sigma ^3/(6V)$, where the hydrodynamic diameter $\sigma$ was introduced in Eq. (33). To simulate bulk systems we use periodic boundary conditions in all three spatial dimensions. To deal with the long-range nature of dipolar interactions, we use the so-called reaction field method^69^. In this method, a cut-off radius $r_{\textrm{RF}}$ is introduced and all interactions for particles closer than $r_{\textrm{RF}}$ are calculated explicitly according to Eq. (31). The far-field contribution is approximated as a magnetic continuum, leading to an additional reaction-field for every particle of the form $\textbf{h}_i^{\textrm{RF}}=\lambda (\sigma /r_{\textrm{RF}})^3\sum _{j\in \mathscr{R}_i}\textbf{u}_j$, where the sum extends over all particles within a sphere of radius $r_{\textrm{RF}}$ centered at particle *i* (and includes particle *i*). We have verified (see e.g. Fig. 9 below) that cut-off values $r_{\textrm{RF}}=5\sigma$ and $8\sigma$ are sufficient to achieve accurate results for the simulation parameters used in our study for volume fractions $\phi \ge 0.05$ and $\phi <0.05$, respectively. The number *N* of MNPs ranges between $N=1000$ for $\lambda \le 2$ and $N=10000$ for $\lambda \ge 4$, and is generally mentioned in the figure captions. A rectangular simulation box is used which is uniaxially extended in the direction of the applied magnetic field. A time step of $\Delta t=0.001\tau _{\textrm{B}}$ is chosen. We ensure proper equilibration before data are collected. Within the stationary regime, data are typically collected over a time interval of $10^4\,\tau _{\textrm{eff}}$.

We have performed several tests to validate our implementation of the model system. For example, we verified that results are independent of the initial configurations and there are no finite-size effects, i.e. results do not change when using larger *N* than reported here. We also reproduced results on cluster sizes available from the literature and several theoretical results in the limit $\phi \rightarrow 0$.

We also verified that the equilibrium magnetization $M_{\textrm{eq}}=M_{\textrm{sat}}\langle \textbf{u}\cdot \hat{\textbf{h}} \rangle _{\textrm{eq}}$ does not depend on *q*. This result is expected – since equilibrium properties should not depend on relaxation times – and provides a further check for proper equilibration of our samples. Moreover, our results for $M_{\textrm{eq}}$ agree quantitatively with earlier simulation results^59^ for $\phi =0.157$ with $\lambda =1$ and $\lambda =2$ (not shown). Figure 9 shows $M_{\textrm{eq}}$ normalized with the saturation magnetization $M_{\textrm{sat}}$ as a function of the dimensionless field strength *h* for different concentrations and dipolar interaction strengths. As reported previously^59^, dipolar interactions increase $M_{\textrm{eq}}$ compared to the non-interacting values. For $\lambda =1$ and 2, this increase is very accurately described by MMF2,

61 $$\begin{aligned} M_{\textrm{eq}} = M_{\textrm{sat}} L_1(h^{\textrm{loc}}), \end{aligned}$$

where the local field $h^{\textrm{loc}}$ is given by Eq. (17) and $L_1(x)$ denotes the Langevin function. Also in agreement with previous observations^48,59^, MMF2 is very accurate up to moderate interaction strengths, but is found to underpredict the magnetization for strong dipolar interactions (here $\lambda =4$). For strong dipolar interactions, the flexible chain model could offer a promising alternative approach.^70^

## Data Availability

The datasets generated during and/or analysed during the current study are available from the corresponding author on reasonable request.
